# The RNA-binding landscapes of two SR proteins reveal unique functions and binding to diverse RNA classes

**DOI:** 10.1186/gb-2012-13-3-r17

**Published:** 2012-03-21

**Authors:** Minna-Liisa Änkö, Michaela Müller-McNicoll, Holger Brandl, Tomaz Curk, Crtomir Gorup, Ian Henry, Jernej Ule, Karla M Neugebauer

**Affiliations:** 1 Max Planck Institute of Cell Biology and Genetics, Pfotenhauerstr. 108, Dresden, 01307, Germany; 2 Laboratory of Molecular Biology, Medical Research Council, Hills Road, Cambridge, CB2 0QH, UK; 3 Faculty of Computer and Information Science, University of Ljubljana, Trzaska cesta 25, Ljubljana, SI-1001, Slovenia; 4 Current address: Australian Regenerative Medicine Institute, Monash University, Wellington Road, Clayton, Melbourne, VIC-3800, Australia

## Abstract

**Background:**

The SR proteins comprise a family of essential, structurally related RNA binding proteins. The complexity of their RNA targets and specificity of RNA recognition *in vivo *is not well understood. Here we use iCLIP to globally analyze and compare the RNA binding properties of two SR proteins, SRSF3 and SRSF4, in murine cells.

**Results:**

SRSF3 and SRSF4 binding sites mapped to largely non-overlapping target genes, and *in vivo *consensus binding motifs were distinct. Interactions with intronless and intron-containing mRNAs as well as non-coding RNAs were detected. Surprisingly, both SR proteins bound to the 3' ends of the majority of intronless histone transcripts, implicating SRSF3 and SRSF4 in histone mRNA metabolism. In contrast, SRSF3 but not SRSF4 specifically bound transcripts encoding numerous RNA binding proteins. Remarkably, SRSF3 was shown to modulate alternative splicing of its own as well as three other transcripts encoding SR proteins. These SRSF3-mediated splicing events led to downregulation of heterologous SR proteins via nonsense-mediated decay.

**Conclusions:**

SRSF3 and SRSF4 display unique RNA binding properties underlying diverse cellular regulatory mechanisms, with shared as well as unique coding and non-coding targets. Importantly, CLIP analysis led to the discovery that SRSF3 cross-regulates the expression of other SR protein family members.

## Background

Gene expression in metazoans is regulated at multiple levels. While investigation of transcriptional regulation by transcription factors has led to a deep understanding of how DNA binding proteins direct RNA polymerases to genes, regulation of RNA processing by RNA-binding proteins is still enigmatic. Hundreds of proteins encoded by metazoan genomes have RNA-binding capacity conferred by specific protein structural domains, such as RNA recognition motifs (RRMs), KH domains and zinc fingers [1]. RNA-binding proteins can change gene expression output at different steps of RNA metabolism, including pre-mRNA splicing, polyadenylation, RNA export, RNA stability, and translation. However, the *in vivo *binding specificity and function(s) of most RNA-binding proteins are not well understood.

SR proteins are a family of seven RNA-binding proteins with a functional repertoire that has expanded to many aspects of RNA metabolism [2,3]. They are concentrated in the nucleus, where they participate in pre-mRNA splicing [4], yet nearly all SR proteins shuttle between the nucleus and cytoplasm. SR protein shuttling activity contributes to their roles in mRNA export, stability and translation [5,6]. SR proteins share a modular structure of one or two RNA recognition motifs (RRMs) at their amino terminus and an arginine-serine-rich RS domain of variable length at the carboxyl terminus. Both domains can directly contact RNA [7], although the RRM appears to determine RNA-binding specificity [5,8,9]. *In vitro *binding specificities have been determined for some SR protein family members [10,11], which bind to 4- to 10-nucleotide long degenerate sequences. Recently, *in vivo *crosslinking was used to define mRNA targets of SRSF1 (also called ASF/SF2); this study identified thousands of SRSF1 target sites, which resembled the sequences derived *in vitro *[12]. Mature mRNAs associated with SRSF3 (SRp20) and SRSF4 (SRp75) were also recently identified and represent functionally distinct mitochondrial ribonucleoproteins (mRNPs) [13]. However, the latter analysis provided information at the gene level and did not identify direct binding sites of SR proteins to RNA targets.

To understand the widespread functions of SR protein family members, the identification of endogenous RNA target sites is required. The development of ultraviolet (UV) crosslinking and immunoprecipitation (CLIP) followed by high-throughput sequencing has made possible the identification of *in vivo *binding sites of RNA-binding proteins in a genome-wide manner [14]. Here we used a modification of the CLIP protocol called iCLIP [15], which allows high-resolution identification of RNA-protein crosslink sites, to investigate the binding specificity and endogenous RNA targets of SRSF3 and SRSF4. We took advantage of our previously developed tagging and stable expression system, in which an enhanced green fluorescent protein (EGFP) tag is inserted at the carboxyl terminus of the SR protein by recombineering of bacterial artificial chromosomes (BACs); due to co-regulation of the endogenous and stably integrated transgenes, the total level of SR protein expression is unchanged in the diploid mouse P19 cells used here [13]. Using the EGFP tag as a universal epitope for iCLIP, we determined *in vivo *binding sites of SRSF3 and SRSF4. Our analysis shows that SRSF3 and SRSF4 bind to distinct sequences and target RNAs, including non-coding RNAs (ncRNAs). The subsequent analysis showed that SRSF3 or SRSF4 binding to these sites conferred regulatory functions in several steps of RNA metabolism in cells, supporting the widespread contribution of SR proteins in gene expression regulation.

## Results

### SRSF3 and SRSF4 bind distinct RNAs

We used the iCLIP method [15] to identify SRSF3 and SRSF4 binding sites genome-wide in mouse P19 cells. SRSF3 and SRSF4 were immunopurified via the EGFP tag encoded on stable transgenes to allow direct comparison of the binding profiles of the two SR proteins [13]. Previous analyses showed that the EGFP-tagged SR proteins recapitulate interactions with nascent RNA and functionally rescue the endogenous proteins [5,13]. Both SRSF3-EGFP and SRSF4-EGFP were specifically and efficiently immunopurified from cell extracts, and SR protein-RNA complexes were isolated after *in vivo *UV crosslinking (Figure S1a, b in Additional file 1). No RNA-protein complexes were detected in cells expressing only nuclear EGFP (EGFP-nuclear localization signal) or in the absence of UV crosslinking (Figure S1b in Additional file 1). In each replicate experiment, SRSF4 showed weaker signal intensity than SRSF3 (Figure S1b in Additional file 1), indicating either lower crosslinking efficiency or fewer RNA targets.

Crosslinked, immunopurified RNA was digested to lengths of 40 to 100 nucleotides, reverse transcribed and prepared for next-generation sequencing [15] (Figure S1c in Additional file 1). The resulting reads, referred to as CLIP-tags throughout the manuscript, were aligned to the mouse mm9 genome assembly. In total, iCLIP produced 1,212,480 and 243,501 unique CLIP-tags for SRSF3 and SRSF4, respectively (Table S1 in Additional file 1). SRSF4 reproducibly yielded fewer sequence reads, in agreement with the lower crosslinking levels observed (Figure S1b in Additional file 1). The EGFP-nuclear localization signal control iCLIP experiments performed in parallel did not produce any detectable PCR products and yielded a total of 2,611 CLIP-tags mapping to the mouse genome. Because the SRSF3 and SRSF4 iCLIPs generated 100- to 1,000-fold more CLIP-tags than the control iCLIP, less than 1% of the detected CLIP-tags could be due to nonspecific crosslinking.

As a first step towards analyzing the RNAs and RNA regions bound by SRSF3 and SRSF4, crosslink sites were identified by mapping to the first nucleotide upstream of the start of each CLIP-tag, as previously described [15]. We determined statistically significant SRSF3 and SRSF4 crosslink sites (33,458 and 10,393, respectively), and identified CLIP-tag clusters with a maximum spacing of 15 nucleotides and containing a significant CLIP-tag count when compared to randomized positions (false discovery rate < 0.05) [15-17]. To test whether the iCLIP captured only the most highly expressed genes, we compared the density of CLIP-tags to our global gene expression data in P19 cells [13]. There was a slight positive correlation between the gene expression level and the density of CLIP-tags within the gene, yet CLIP-tags were identified in genes at the whole range of gene expression (Figure S1d in Additional file 1).

**Figure 1:**
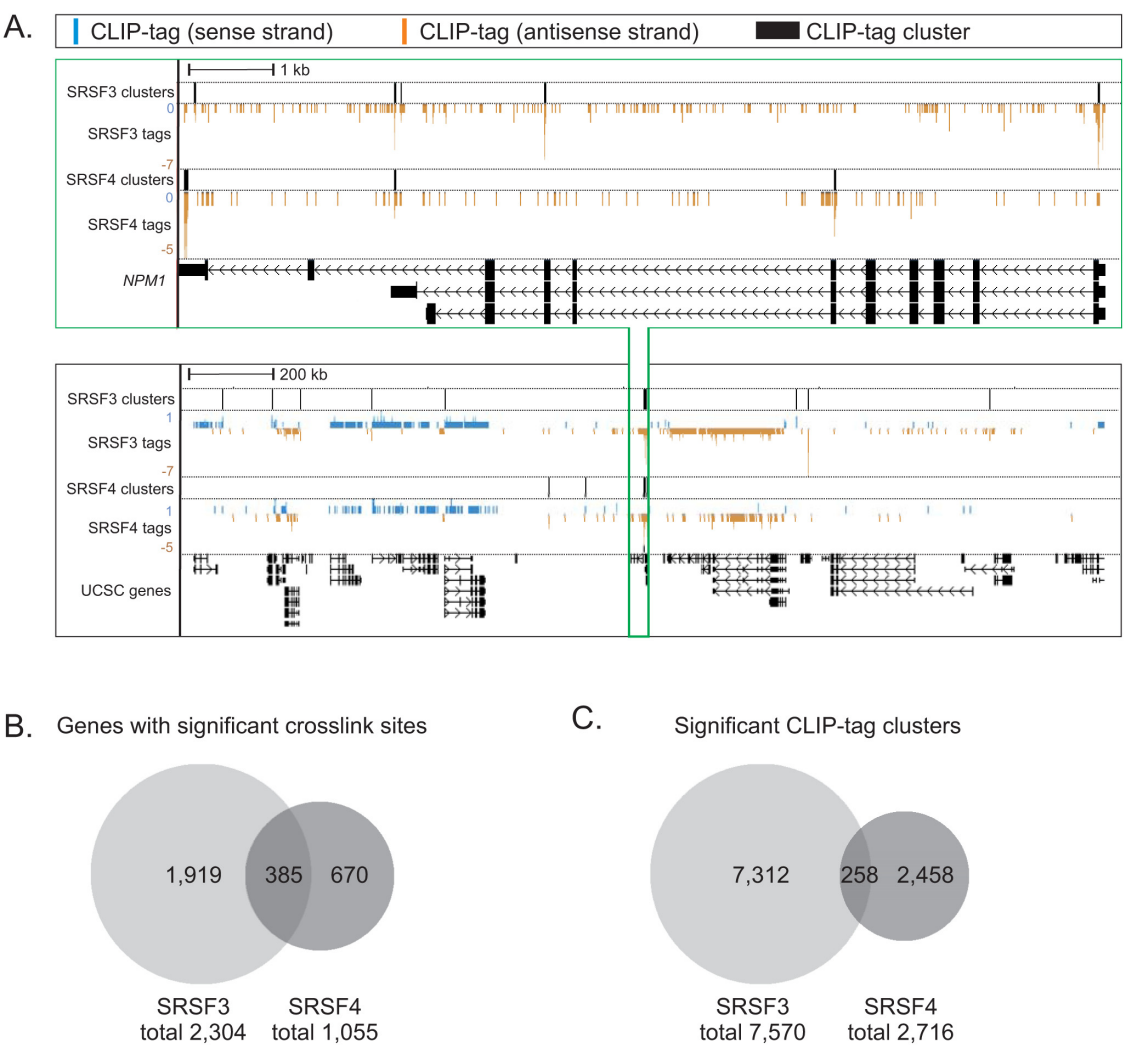
**SRSF3 and SRSF4 CLIP-tags cluster to distinct positions in mouse RNAs**. **(a) ***NPM1 *gene (green box) and the surrounding approximately 3 MB region in chromosome 11 (black box) with SRSF3 and SRSF4 CLIP-tags and clusters. The numbers on the left represent the number of CLIP-tags within the window. The sense strand is marked in blue and the antisense strand in orange. Note that the genes in the antisense strand run from right to left. **(b) **Comparison of annotated genes with significant SRSF3 or SRSF4 crosslink sites (false discovery rate < 0.05). **(c) **Comparison of significant SRSF3 and SRSF4 CLIP-tag clusters (overlap of clusters ≥ 15 nucleotides).

Examination of SRSF3 and SRSF4 CLIP-tag clusters indicated that multiple reads were detected in limited RNA regions. The same transcript could display crosslinking to both SR proteins, albeit in different regions of the transcript, as exemplified by the *NPM1 *gene that contained CLIP-tag clusters for both SRSF3 and SRSF4 mapping to distinct exons (Figure 1a). Also at the chromosome level, a large proportion of the CLIP-tags and clusters were non-overlapping (Figure 1a; Figure S2 in Additional file 1). Significant crosslink sites were detected in 2,304 genes for SRSF3 and 1,055 genes for SRSF4, of which 83.3% and 83.2% were protein-coding, respectively. A list of genes with significant crosslink sites is provided in Additional file 2. These numbers are likely to be underestimates because our sequencing has not reached saturation. In agreement with our recent analysis showing that SRSF3 and SRSF4 associate with distinct mRNAs [13], the identity of the target RNAs bound by SRSF3 and SRSF4 only partially overlapped (Figure 1b). An even smaller overlap between SRSF3 and SRSF4 CLIP-tag clusters, rather than genes, was observed (compare Figure 1b and 1c), strongly suggesting differential RNA-binding specificities of SRSF3 and SRSF4.

### Consensus binding motif of SRSF3 and SRSF4

**Figure 2:**
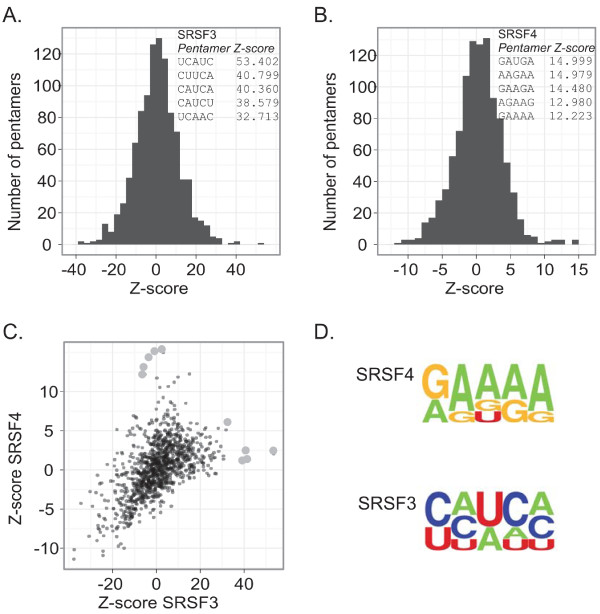
***In vivo *binding specificity of SRSF3 and SRSF4**. **(a, b) **The frequency distribution of SRSF3 (a) and SRSF4 (b) pentamer Z-scores. The Z-score was calculated relative to randomized genomic positions by shuffling the crosslink positions 100 times within the genes. Five pentamers with highest Z-scores are shown. **(c) **Correlation of SRSF3 and SRSF4 pentamer Z-scores. The top five pentamers presented in (a, b) are marked as larger light grey dots. **(d) **Consensus motifs were derived from the top pentamers shown in (a, b).

The *in vivo *binding specificities of SRSF3 and SRSF4 are unknown. The differences in the CLIP-tag cluster sites suggested that each of the two SR proteins binds to a distinct RNA sequence. To address this directly, we used the data to derive *in vivo *binding motifs for SRSF3 and SRSF4 by analyzing enriched pentamer sequences around the crosslink sites. To calculate a Z-score for each pentamer, iCLIP positions were randomized within the same regions. The pentamer enrichment analysis showed that SRSF3 and SRSF4 identify distinct sequence motifs (Figure 2). The top five pentamers for SRSF3 (Figure 2a) were in close agreement with the core SELEX (systemic evolution of ligands by exponential enrichment) motif determined *in vitro *[18,19]. SELEX has not been performed on SRSF4; interestingly, the SRSF4 top five pentamers (Figure 2b) were similar to one sequence (GAAGGA) previously shown to be an SRSF4 binding site in bovine papilloma virus pre-mRNA [20]. The SRSF3 binding motif was CU-rich excluding Gs, whereas SRSF4 bound to GA-rich sequences excluding Cs (Figure 2d). These results are consistent with the largely non-overlapping SRSF3 and SRSF4 crosslink sites and clusters (Figure 2c, and see above).

### SRSF3 and SRSF4 bind to coding and non-coding RNAs

**Figure 3:**
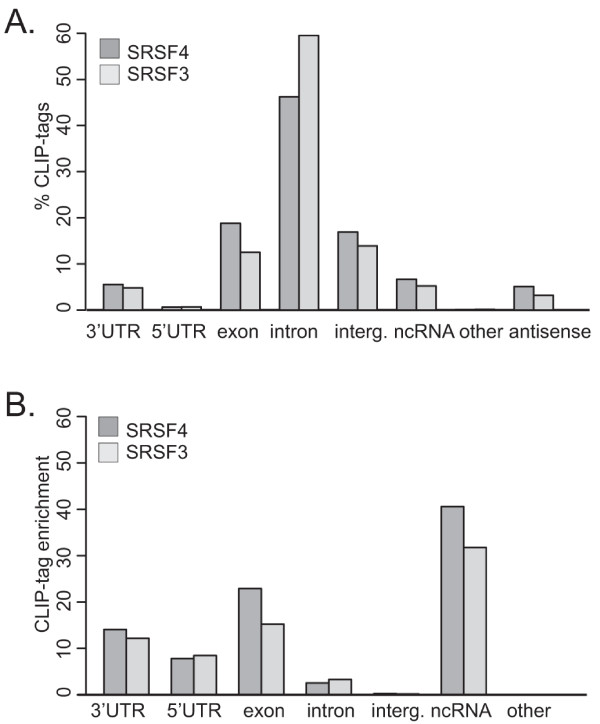
**Distribution of SRSF3 and SRSF4 CLIP-tags within RNA classes and transcript regions**. **(a) **The proportion of CLIP-tags that mapped to different RNAs relative to the total number of CLIP-tags. **(b) **The fold enrichment of CLIP-tag density (the number of CLIP-tags divided by the length of each RNA feature) in different RNAs relative to the average CLIP-tag density in the genome.

Which categories of RNA and which functional RNA regions are bound by SR proteins? Analysis of the frequency with which SRSF3 and SRSF4 CLIP-tags were mapped to genes and gene regions revealed their common propensity to bind exons and introns in protein-coding genes (Figure 3a; Table S3 in Additional file 1). The high proportion of intronic CLIP-tags detected clearly reflects the fact that mammalian introns are much longer than exons; when the frequency of CLIP-tags was normalized to the length of the RNA region (Figure 3b), both SRSF3 and SRSF4 CLIP-tags were more highly enriched in exons than in introns. SR protein interactions with exons could reflect activities either in pre-mRNA splicing or in mRNPs after splicing (see below).

**Figure 4:**
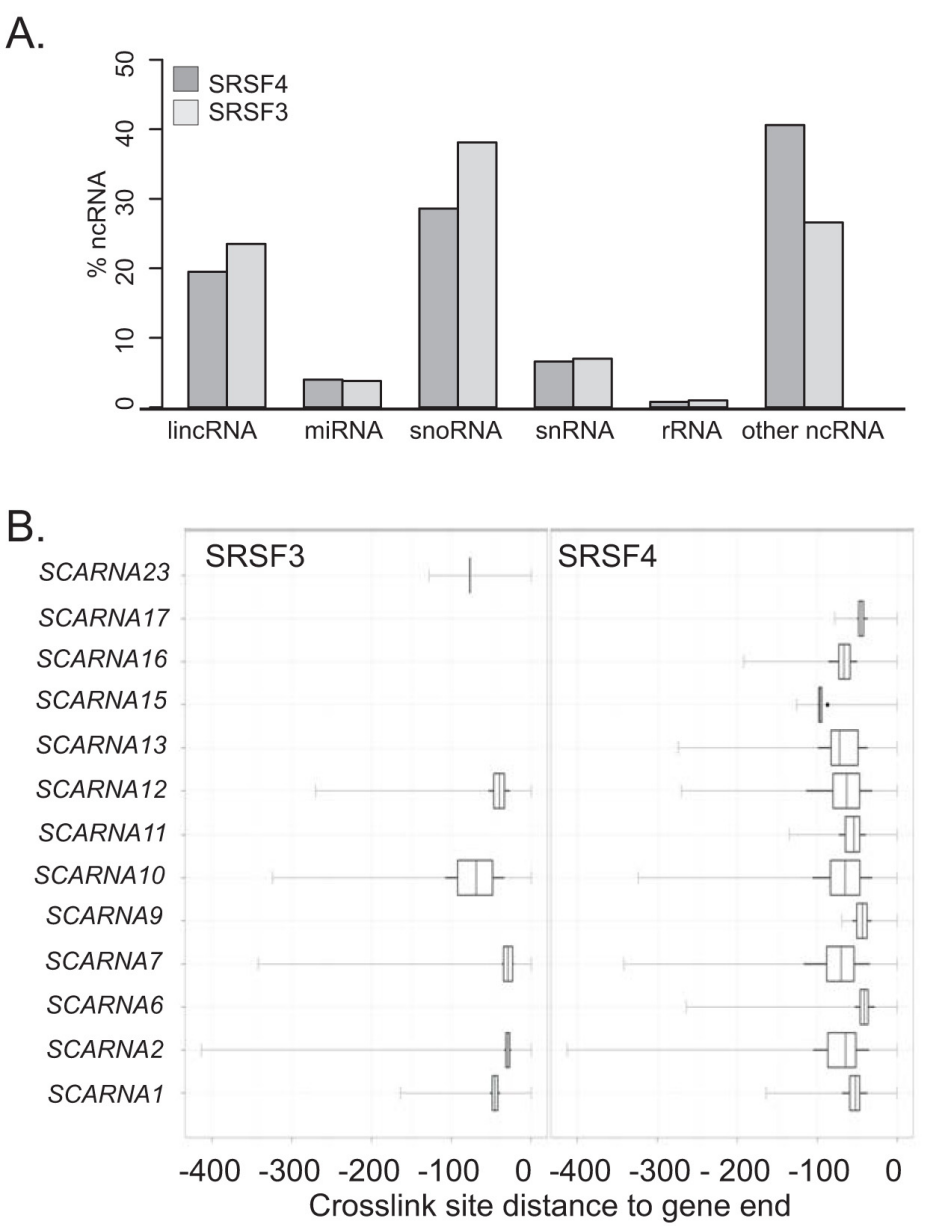
**ncRNAs with SRSF3 and SRSF4 crosslink sites**. **(a) **The distribution of crosslink sites within the ncRNA subclasses. **(b) **The position of the SRSF4 CLIP-tag clusters relative to the scaRNA 3' end. 'Other ncRNAs' are processed transcripts with no known ORF or function.

The highest density of CLIP-tags was detected in ncRNAs (Figure 3b). Overall, 319 and 141 ncRNAs had SRSF3 and SRSF4 CLIP-tag clusters, respectively. The most abundant ncRNA classes with CLIP-tags were long ncRNAs (lincRNAs) and small nucleolar RNAs (snoRNAs) (Figure 4a). Similar to SRSF1 and TDP-43 [12,21], SRSF3 and SRSF4 crosslinked to the lincRNA *MALAT1 *(aka *NEAT2*; Figure S3a in Additional file 1) that is enriched in nuclear speckles [22]. In addition, another speckle-localized ncRNA, *7SK *[23], had abundant SRSF3 and SRSF4 CLIP-tag clusters (data not shown). An especially large proportion of ncRNAs with SRSF3 and SRSF4 crosslink sites belonged to snoRNAs, a class of small RNAs that guide RNA modifying enzymes [24]. Intriguingly, small Cajal body-specific RNAs (scaRNAs), a subclass of snoRNAs, were enriched in SRSF4 CLIP-tag clusters. SR protein binding could not be correlated with known elements within scaRNAs because the scaRNAs identified included those with H/ACA boxes alone, C/D boxes alone, and a combination of H/ACA and C/D boxes. The specificity of SR protein binding to this group of scaRNAs was investigated in two ways. First, we asked whether binding was biased to any particular region of the scaRNAs. Figure 4b shows that binding sites were localized near scaRNA 3' ends (Figure 4b; Figure S3a in Additional file 1). Second, the CLIP-tag clusters within the scaRNAs were used to determine a consensus binding motif independent of the global pentamer analysis. Multiple alignment of the CLIP-tag cluster regions using the MEME (Multiple Em for Motif Elicitation) algorithm identified a consensus sequence element (Figure S3c in Additional file 1) that was found in all scaRNAs with SRSF4 CLIP-tag clusters. The motif was GA-rich, similar to the pentamer motif determined for all crosslink sites with the exception that Cs were occasionally observed. This independent derivation of a binding sequence similar to the globally derived consensus indicates that SRSF4 binding to scaRNAs is specific.

### SRSF3 and SRSF4 bind to intronless histone mRNAs

**Figure 5:**
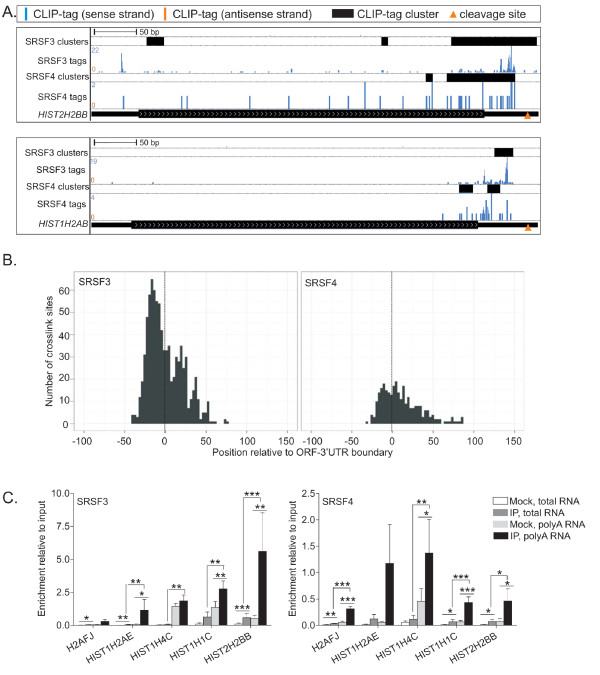
**SRSF3 and SRSF4 bind to numerous intronless histone mRNAs at a consistent position**. **(a) **SRSF3 and SRSF4 CLIP-tags and clusters in *HIST2H2BB *and *HIST1H2AB *genes. Labels as in Figure 1a. The orange arrowheads mark the mRNA 3' end cleavage site. **(b) **Mapping of SRSF3 (left panel) and SRSF4 (right panel) crosslink sites to the ORF-3' UTR boundary of histone mRNAs. The position 0 marked with a dotted line represents the ORF-3' UTR boundary. **(c) **Cytoplasmic levels of histone mRNAs associated with SRSF3 or SRSF4 determined by UV-RNA immunoprecipitation and reverse transcription quantitative PCR. To prime the reverse transcription reactions, hexamers were used to detect total and oligo-dT to detect polyadenylated histone mRNAs. Data are presented relative to the input sample. Mock is the non-immune control. *P < 0.05, **P < 0.01, ***P < 0.001 (Student's unpaired *t*-test, *n *= 3-6). Error bars are standard deviation. IP, immunoprecipitation.

SRSF3 and SRSF4 binding sites were found in intronless protein-coding genes, likely reflecting SRSF3 and SRSF4 participation in regulatory events other than splicing. In particular, SRSF3 and SRSF4 CLIP-tag clusters were detected within histone genes: 73.8% of the mouse histone genes annotated in [25] had SRSF3 clusters and 47.7% had SRSF4 clusters (Figure 5a; Figure S4a in Additional file 1). This was also reflected in the enriched Gene Ontology (GO) terms where categories related to chromatin and nucleosome assembly were present (Table S4 in Additional file 1). The SRSF3 and SRSF4 CLIP-tag clusters were located at the boundary between ORF and 3' UTR and/or within the 3' UTR of histone mRNAs (Figure 5b). The CLIP-tag clusters were located just upstream of conserved stem-loops that occur 14 to 50 nucleotides downstream of the ORF (Figure 5a); these stem loops specify the sites of endonucleolytic cleavage of replication-dependent histone mRNAs and therefore define their 3' ends [26].

SRSF3 was previously shown to promote the export of histone *H2A *reporter mRNAs via a 22-nucleotide transport element within the coding region of *H2A *mRNAs, to which SRSF3 bound and recruited the mRNA export factor TAP [27,28]. In our study, however, most SRSF3 and SRSF4 CLIP-tag clusters in histone *H2A *family mRNAs were found outside this 22-nucleotide transport element (Figure 5a, b; Figure S4a in Additional file 1). Furthermore, most SRSF3 and SRSF4 crosslink sites were present in mRNAs of histone families other than *H2A*, which do not contain the transport element (Additional file 2). Interestingly, SRSF3 and SRSF4 binding sites identified here are similar to those reported in another study that characterized export factor-binding sites in histone mRNAs [29].

SR proteins also promote polyadenylation in some contexts [30,31]. We found this intriguing in the context of the histone mRNA targets because several recent studies have shown that a significant pool of histone mRNAs undergo polyadenylation instead of 3' end cleavage [32-36]. To validate the association of SRSF3 and SRSF4 with histone mRNAs and to investigate polyadenylation, we adopted an RNA immunoprecipitation (RIP) assay from UV crosslinked cell extracts (UV-RIP); the immunoprecipitation was carried out from a cytoplasmic fraction in order to avoid contamination by genomic DNA that would later influence results obtained by reverse transcription quantitative PCR (RT-qPCR) (Figure S4b in Additional file 1). Both total and polyadenylated histone mRNA levels were measured in the SRSF3 and SRSF4 immunoprecipitates, using either random hexamers or oligo-dT as reverse primers. Figure 5c shows that both SR proteins immunoprecipitated histone mRNAs significantly above mock immunoprecipitates, irrespective of which reverse primer was used. Compared to input, detection of histone mRNAs was more robust when oligo-dT reverse primers were used, suggesting that SRSF3 and SRSF4 preferentially bind polyadenylated histone mRNAs. The detection of SRSF3 and SRSF4 bound to polyadenylated histone mRNAs in the cytoplasmic fraction suggests that both SR proteins may be involved in histone mRNA 3' end formation, export, and/or translation.

### SRSF3 and SRSF4 make diverse contacts with exons and introns

**Figure 6:**
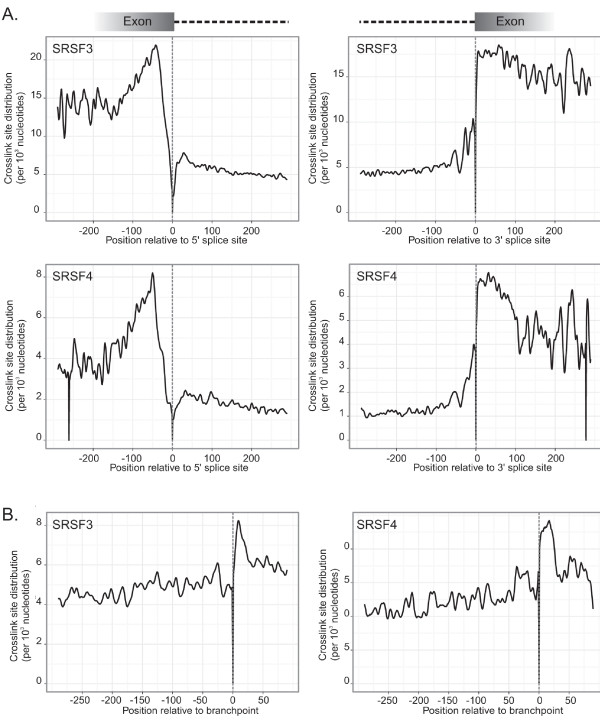
**SRSF3 and SRSF4 contact exons and introns**. **(a) **SRSF3 and SRSF4 crosslink sites mapped around the 5' and 3' splice sites. The position 0 (dotted line) represents the indicated 5' or 3' splice site; the y-axis represents normalized crosslink sites per 10^3 ^nucleotides. The normalization is based on the length distribution of exons and introns (Figure S5 in Additional file 1). The data were smoothed using a Gaussian window (half-width of the window = 5). **(b) **SRSF3 and SRSF4 crosslink sites mapped to predicted mouse branch points. The position 0 (dotted line) represents the branch point nucleotide. Smoothing as in (a).

Because SR proteins are known to regulate pre-mRNA splicing, we wondered whether the crosslink sites were correlated with particular locations within introns and/or exons. Data from *in vitro *studies suggest that SR proteins bind pre-mRNAs primarily within exons and thereby recruit spliceosomal components to adjacent 5' and 3' splice sites [37]. Therefore, crosslink sites were mapped to exon-intron and intron-exon boundaries. Variability in exon and intron length genome-wide leads to an apparent abundance of CLIP-tags close to the junctions (Figure S5a in Additional file 1). Therefore, we established a normalization factor derived from the length distribution of exons and introns to correct for these differences (Figure S5b in Additional file 1). Mapping of normalized crosslink sites showed exonic enrichment of SRSF3 and SRSF4 crosslink sites, which were most pronounced within 100 nucleotides of both 5' and 3' splice sites (Figure 6a). Peaks of SRSF3 and SRSF4 binding approximately 70 nucleotides upstream of 5' splice sites were more prominent than peaks observed downstream of 3' splice sites. Note that we did not map sequences falling onto exon-exon junctions, which explains the drop in crosslinking immediately upstream of 5' splice sites. Because SR proteins bind mRNA as well as pre-mRNA, it seems logical that exon sequences are overrepresented in the experimental data compared to intron sequences. However, similar patterns of enrichment in exons were observed when the pentamer motifs alone were considered (Figure 2; Figure S5c in Additional file 1), suggesting that the observed exon bias reflects the distribution of binding sequences within target RNAs. Interestingly, we noticed a peak of crosslink sites approximately 30 nucleotides upstream of 3' splice sites (Figure 6a). This corresponds to the approximate position of branch points in mammalian introns. However, the actual position of the branch point varies relative to the 3' splice site, with the longest observed distance of 400 nucleotides [38]. Therefore, crosslink sites were mapped to predicted mouse branch points [39]. This mapping indicated that SRSF3 and SRSF4 bind at or slightly downstream of the branch point nucleotide (Figure 6b). In conclusion, SRSF3 and SRSF4 preferentially contact exonic sequences, especially upstream of 5' splice sites; they also interact with branch points as suggested by two previous studies [7,40], consistent with the model that SR proteins regulate splicing by contacting pre-mRNA in different functional regions.

### SRSF3: a regulator of splicing factors

**Figure 7:**
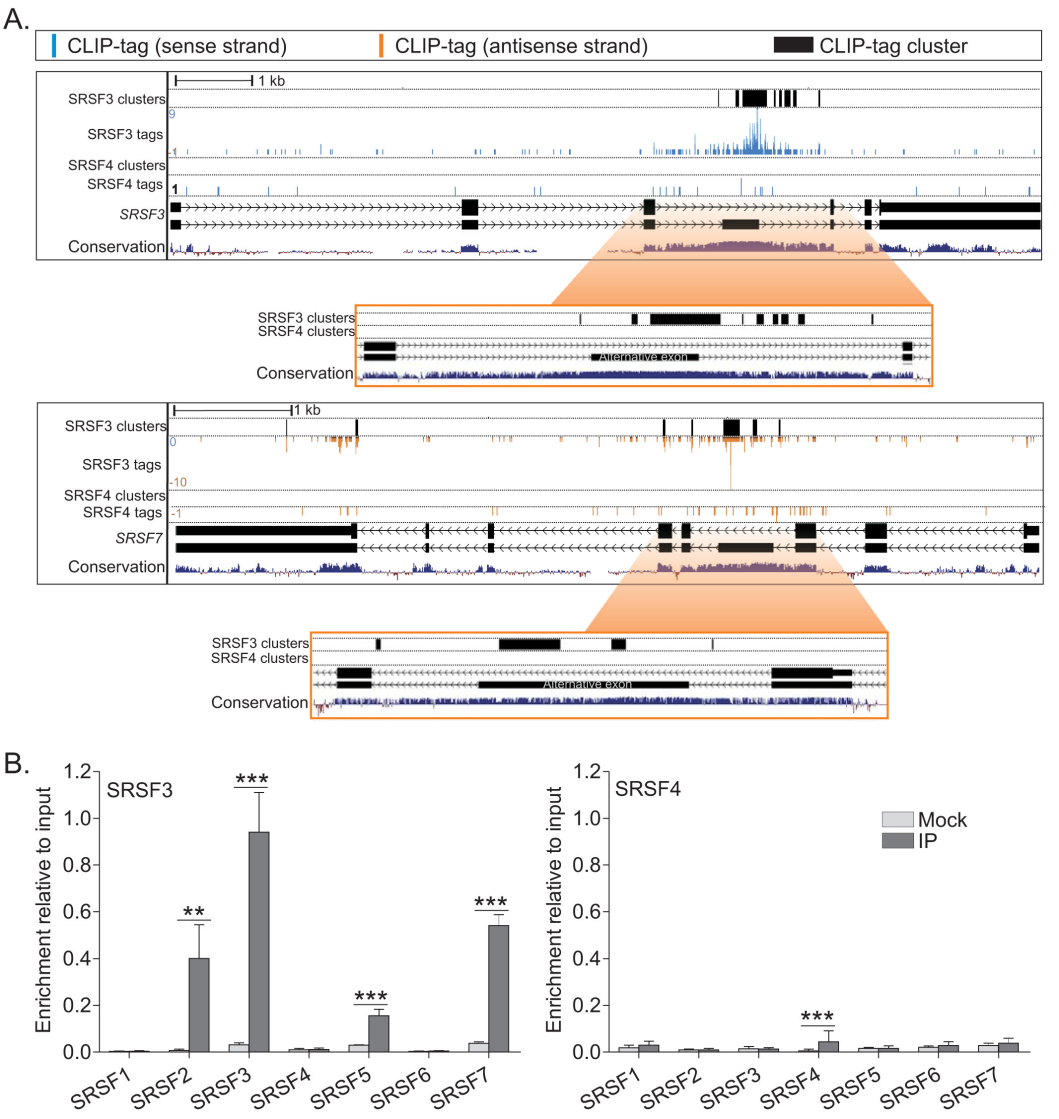
**SRSF3 binds to poison cassette exons in SR proteins**. **(a) **SRSF3 and SRSF4 CLIP-tags and clusters around the alternative cassette exon of *SRSF3 *and *SRSF7 *genes. Labels as in Figure 1a. The zoom in represents the ultraconserved regions identified in [42,43]. Note that the genes in the antisense strand run from right to left. **(b) **The enrichment of mRNAs encoding different SR protein family members after UV crosslinking and SRSF3 or SRSF4 immunoprecipitation (IP). To prime the RT reactions, hexamers were used. Data are presented relative to the input sample. IP is the specific immunoprecipitation and mock is the non-immune control. *P < 0.05, **P < 0.01, ***P < 0.001 (Student's unpaired *t*-test, *n *= 3-6). Error bars are standard deviation.

The notion that different splicing factors might regulate transcripts with similar functions, creating an expression module regulated by splicing, has intrigued the field for decades. We therefore asked about the functional identity of SRSF3 and SRSF4 protein-coding targets. Similar to our previous findings by RIP-chip [13], GO analysis of the protein-coding genes with significant SRSF3 and SRSF4 crosslink sites revealed functions related to nucleic acid binding and RNA processing as the most enriched GO terms for both SRSF3 and SRSF4 (Table S4 in Additional file 1). SRSF3 binding sites were especially enriched within genes encoding components of RNP complexes, including splicing factors (Table S5 in Additional file 1). SRSF3 crosslink sites were found within the genes encoding other SR proteins, as well as in proteins of heterogeneous nuclear ribonucleoprotein complexes and components of the core splicing machinery. SRSF3 is known to strictly regulate its own expression through an inclusion of a premature termination codon (PTC)-containing cassette exon, which is referred to as a 'poison cassette exon' because it leads to transcript degradation by nonsense-mediated decay (NMD) [13,41]. Poison cassette exons occur in all SR protein family members and are ultraconserved among species [42,43]. The inclusion of the alternative cassette exon or intron retention leads to the introduction of a PTC in the SR protein mRNA in every case. Indeed, SRSF3 and SRSF4 CLIP-tag clusters were detected in the *SRSF3 *and *SRSF4 *autoregulatory cassette exons, respectively (Figure 7a, top panel; Figure S6, bottom panel, in Additional file 1).

To date, it has been assumed that poison cassette exons are recognized by the gene's own protein product, in an auto-regulatory feedback loop (see above). Intriguingly, the SRSF3 CLIP-tag clusters were also found in the NMD-associated exons or introns of three heterologous SR protein-encoding genes, *SRSF2, SRSF5 *and *SRSF7 *(Figure 7a; Figure S6 in Additional file 1). In contrast, SRSF4 CLIP-tag clusters were found only in the poison cassette exon of its own pre-mRNA. We sought to validate the specificity of these interactions by UV-RIP. SRSF3 specifically immunoprecipitated *SRSF2, SRSF3, SRSF5 *and *SRSF7 *(pre-)mRNAs, whereas SRSF4 only immunoprecipitated significant levels of its own (pre-)mRNA (Figure 7b). These data validate the specificity of SRSF3 interactions with heterologous transcripts encoding SR protein family members in the manner indicated by iCLIP; note that low recovery of some transcripts may be due to the short half-lives of the bound, PTC-containing messages.

**Figure 8:**
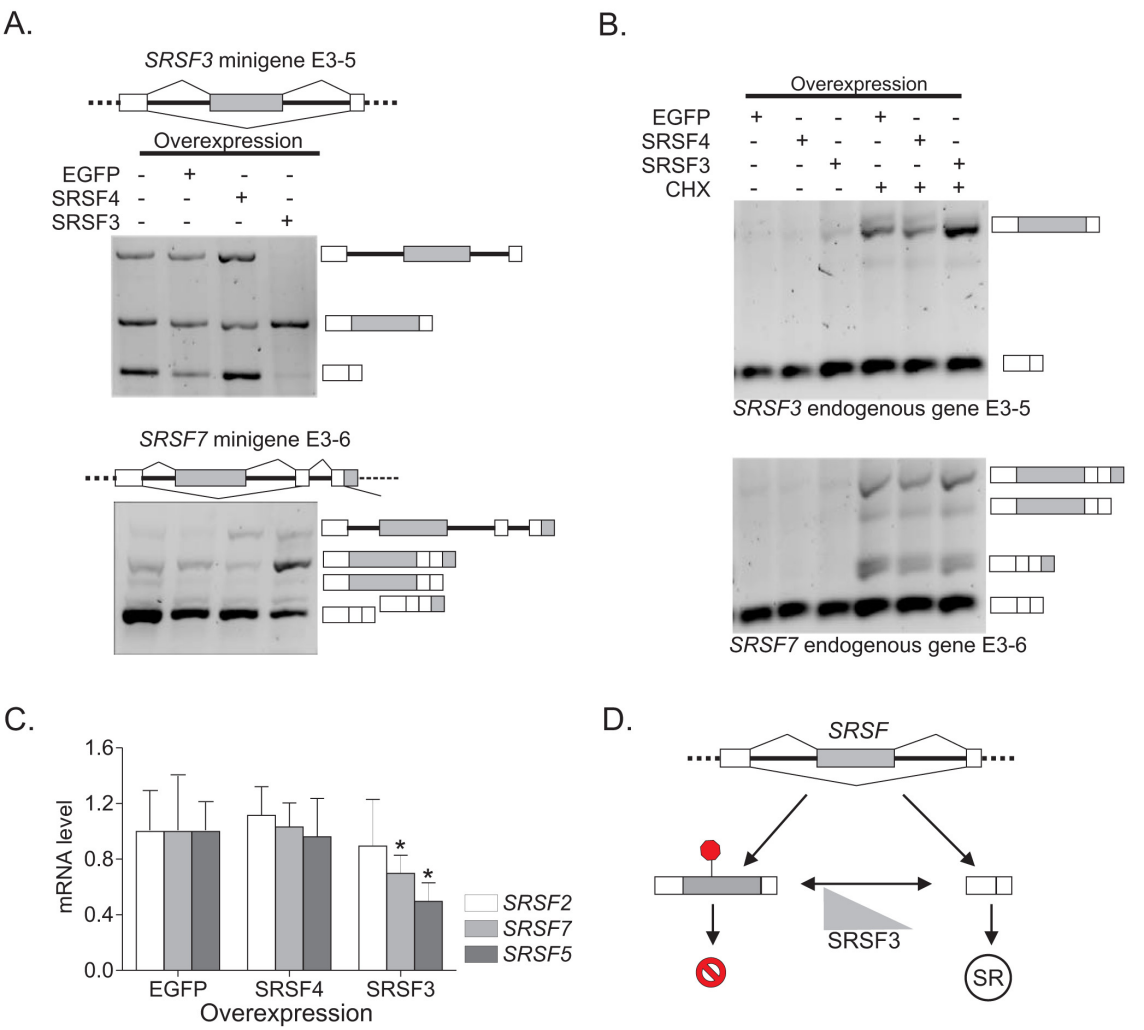
**SRSF3 controls the level of SR proteins through splicing regulation**. **(a) **The splicing products of *SRSF3 *and *SRSF7 *minigenes determined after 24-hour over-expression of SRSF3, SRSF4 or EGFP (control). The alternative exons are marked with light grey. **(b) **The splicing products of endogenous *SRSF3 *and *SRSF7 *after inhibition of NMD by a 3-hour treatment with cycloheximide (CHX). **(c) **The expression level of endogenous, mature *SRSF2, SRSF5 *and *SRSF7 *mRNAs upon EGFP, SRSF3 or SRSF4 overexpression (24 hours) as measured by RT-qPCR. *P < 0.05 (one-way ANOVA). Error bars are standard deviation. *ACTB *was used as the reference gene. **(d) **Schematic showing how SRSF3 controls the levels of other SR protein family members through alternative splicing. The inclusion of a poison cassette exon harboring a premature termination codon (PTC, red stop sign) leads to RNA degradation through NMD.

The presence of SRSF3 CLIP-tag clusters in heterologous SR protein-encoding transcripts could indicate that SRSF3 either positively or negatively regulates poison-cassette exon usage. If so, we would predict that SRSF3 levels in cells should affect the alternative splicing and ultimately expression levels of the three target SR protein transcripts identified. To test this directly, minigenes including the genomic regions around SRSF3 CLIP-tag clusters were constructed for *SRSF2, SRSF3, SRSF5 *and *SRSF7 *(Figure 8a; Figure S7C in Additional file 1). Efficient SRSF3 or SRSF4 protein over-expression and knockdown was achieved by transfection of cDNA expression constructs and RNA interference, respectively (Figure S7a in Additional file 1). Under these conditions, the splicing patterns of the minigene-encoded transcripts were analyzed, using vector-specific primers for RT-PCR. Figure 8a shows that over-expression of SRSF3 led to a marked increase in poison cassette exon inclusion for both the *SRSF3 *and *SRSF7 *minigenes. Upon SRSF3 knockdown, this pattern was reversed (Figure S7b in Additional file 1). Similarly, SRSF3 over-expression led to alternative splicing changes for the *SRSF2 *and *SRSF5 *minigenes, leading to increased poison cassette usage and/or intron retention (Figure S7c in Additional file 1). Importantly, SRSF4 over-expression or knockdown did not detectably alter splicing patterns (Figure 8a; Figure S7b, c in Additional file 1).

The alternative splicing events regulated by SRSF3 documented above predict that the transcripts regulated by SRSF3 - namely *SRSF3 *itself as well as *SRSF2, SRSF5 and SRSF7 *- will undergo degradation through NMD when SRSF3 is over-expressed. To test this, the NMD pathway was inhibited by treating the cells with cycloheximide (CHX) [44]. The use of CHX as a tool also enabled us to investigate the alternative splicing outcome of endogenous transcripts. Figure 8b shows that CHX treatment leads to detection of the otherwise highly unstable endogenous poison cassette exon-containing SR protein transcripts that increase in abundance upon SRSF3 over-expression. Another prediction of these findings is that the steady-state levels of heterologous SR protein transcripts will depend on SRSF3 levels. Through measurement of target mRNA levels by RT-qPCR, we show that *SRSF5 *and *SRSF7 *mRNA levels decrease significantly in cells over-expressing SRSF3 (Figure 8c). Upon CHX treatment, mRNA levels recovered to those of the control (Figure S7d in Additional file 1). Taken together, the data indicate that SRSF3 specifically binds not only its own but other SR protein transcripts and the binding leads to alternative splicing changes that increase the occurrence of PTCs, which in turn target the expressed transcripts for degradation through the NMD pathway. Thus, SRSF3 regulates the expression of its own mRNA and the mRNAs encoding three other SR protein family members (Figure 8d). This cross-regulation by SRSF3 and the observation that many other RNA binding proteins may similarly be regulated by SRSF3 (Table S5 in Additional file 1) raises the possibility that SRSF3 is a master regulator of the transcriptome acting through a network of feedback mechanisms.

## Discussion

Here we used iCLIP to investigate the RNA-binding landscape of two SR proteins, SRSF3 and SRSF4, in mouse cells. The value of this study is enhanced by the global comparison of RNA targets and binding sites for two members of this prominent family of RNA binding proteins with a variety of known roles in gene expression. Through detailed analysis of the transcripts and transcript regions bound by SRSF3 and SRSF4, we provide evidence for previously unknown functions of these highly conserved RNA binding proteins. Here we discuss our findings in the context of five major conclusions.

First, SRSF3 and SRSF4 exhibit largely non-overlapping binding sites and RNA targets, indicating that SRSF3 and SRSF4 regulate specific sets of genes through their interaction with different RNA sequences. This finding is consistent with the previous finding that SRSF3 and SRSF4 are present in non-overlapping mRNPs *in vivo *[13], although the previous study did not examine direct binding sites. The genome-wide CLIP data provided large numbers of binding sites, enabling us to derive consensus *in vivo *binding sequences. We show that SRSF4 binds a consensus GA-rich sequence. The CU-rich SRSF3 *in vivo *consensus binding sequence is similar to that derived *in vitro *by SELEX [18,19], indicating the validity of the use of SELEX to understand binding specificity alone. However, bioinformatic analysis of SELEX sequences does not permit the identification *in vivo *RNA targets because the shortness and degeneracy of consensus sequences leads to over-representation within the transcriptome [45]. The identification of *in vivo *targets, accomplished here by CLIP, allowed us to further investigate the identified RNA classes and RNA regions bound.

Second, an important class of RNAs bound by both SRSF3 and SRSF4 were ncRNAs. SRSF3 and SRSF4 crosslinked to multiple sites along the lincRNA *MALAT1*, which is enriched in nuclear speckles and interacts with a subset of SR proteins, including SRSF3 [22]. Other splicing factors localized in nuclear speckles, such as TDP-43 and SRSF1, also bind to *MALAT1 *[12,21]; it appears, therefore, that *MALAT1 *interaction is common among RNA-binding proteins in nuclear speckles. Furthermore, SRSF3 and SRSF4 interacted with *7SK*, another ncRNA localized to speckles [23] but not with the paraspeckle ncRNA component *NEAT1 *[46]. One especially overrepresented group of short ncRNAs with SRSF3- and SRSF4-binding sites was snoRNAs. We show that a subset of snoRNAs, the scaRNAs, are prominent targets of SRSF4 with binding sites near their 3' ends. SR proteins are likely required for the splicing of all introns, including those containing snoRNAs. The position of the snoRNAs, including scaRNAs, within the host intron is critical for snoRNA processing, implying that the spliceosome is actively involved in the release of snoRNAs from the debranched intron [47]. It remains to be investigated whether SR proteins are required for snoRNA processing from host introns. An interesting possibility is that SR proteins interact within the snoRNA-derived short RNAs and co-regulate alternative splicing [48]. Because the functions of many ncRNAs are currently poorly understood, it will be interesting to determine whether ncRNAs have functions as co-regulators of splicing.

Third, we provide evidence that SRSF3 and SRSF4 bind many intronless genes, further supporting their role as regulators of gene expression independent of pre-mRNA splicing [2]. Surprisingly, we show that SRSF3 and SRSF4 display clusters of binding sites at the ORF-3' UTR junction of the vast majority of histone mRNAs. This binding region was previously shown to be important for the export of histone H2A mRNAs in *Xenopus *oocytes [29]. UV-RIP experiments indicate that both SR proteins bind preferentially to polyadenylated histone mRNAs and that these mRNP complexes are detectable in the cytoplasm. Replication-dependent histone mRNAs are mainly processed by 3' end cleavage [26], and are exported from the nucleus via the stem loop binding protein (SLBP) [32]. It was known that histone mRNAs become polyadenylated when the 3' end cleavage machinery is compromised [26]. However, recent high-throughput sequence analyses of human and mouse mRNAs identified significant pools of polyadenylated mRNAs encoding all four core histones even when the 3'-end processing machinery is functional [35,36]. Thus, expression of polyadenylated histone mRNAs appears to be physiologically important. If polyadenylated histone transcripts fail to bind SLBP, they may require SRSF3 and SRSF4 for export from the nucleus; both SRSF3 and SRSF4 shuttle to the cytoplasm and SRSF3 binds the mRNA export receptor TAP [5,28]. It will be of interest to determine how SRSF3 and SRSF4 regulate histone mRNA 3' end formation, export, or both.

Fourth, intron-containing protein-coding transcripts were a major class of SRSF3 and SRSF4 targets. SR proteins are thought to bind primarily to exonic splicing enhancers, where they influence recognition of adjacent 5' and 3' splice sites [49]. The pattern of binding to exon-intron junctions resembles that observed for SRSF1 [12]; abundant binding within the exon bodies dropped sharply towards exon-intron boundaries. These data agree with the current concept that SR proteins promote adjacent splice site recognition. However, our data show that neither SRSF3 nor SRSF4 binding is limited to exons; instead, a large number of binding sites are found in introns and 3' UTRs. Crosslinking to exonic regions should be overrepresented in CLIP, owing to the low abundance of pre-mRNA in living cells. It is remarkable, therefore, that 65% of SRSF3 and 52% of SRSF4 binding sites were located in introns. SRSF3 was shown to regulate splicing by binding to an intronic splicing enhancer [50], providing precedence for SRSF3 splicing regulatory function via intronic regulatory elements. Intriguingly, SRSF3 and SRSF4 binding was also detected at and around branch-point sequences within introns. *In vitro *studies suggested that SRSF1 is first recruited to an exonic splicing enhancer and the RS domain subsequently contacts the branch point [7,40]. The present study does not distinguish whether the RRM or the RS domain of SRSF3 and/or SRSF4 binds to the branchpoint. Although some correlation between binding to the branch point and to the downstream exon was observed, a strict requirement for binding to both downstream exonic splicing enhancer and the branch point was not detected. Therefore, SR proteins may have exon-independent functions as proposed previously [51].

Finally, we have discovered that SRSF3 cross-regulates the expression of other SR protein family members. Autoregulation, in which SRSF1 and SRSF3 modulate expression of their own messages via inclusion of a PTC-containing exon, was previously known [41,52]. This activity has been attributed to ultraconserved regions within alternative cassette exons and retained introns that introduce a PTC in mRNAs encoding SR proteins [42,43]. Here we identify SRSF3 binding sites in the mRNAs encoding three additional members of the SR protein family and show that short-term overexpression of SRSF3 led to robust effects on the splicing and expression of four SR protein family members (*SRSF2, SRSF3, SRSF5*, and *SRSF7*). This reveals an unexpected role of SRSF3 in cross-regulating expression of other SR protein family members (Figure 8d). SRSF3 binding sites were also abundant in mRNAs encoding other splicing factors and spliceosomal components. These data, together with the observation that SRSF3 binds numerous transcripts encoding RNA binding proteins, underscore the notion that the splicing machinery is under strict cellular control and indicate that SRSF3 is a key regulator of RNA metabolism.

## Conclusions

Our global analysis of SRSF3 and SRSF4 shows that these SR proteins are multifunctional RNA binding proteins interacting with distinct classes of RNA. Initially identified as splicing factors, SRSF3 and SRSF4 regulate constitutive and alternative exons by binding to both exonic and intronic positions. SRSF3 in particular seems to function as a master regulator of splicing machinery expression through its activities in alternative splicing. However, SRSF3 and SRSF4 also interact with RNAs that are not processed by the spliceosome, suggesting a network of interactions that control cellular programs of gene expression. In addition to the numerous functions already assigned for SR proteins, interactions with different RNA classes, including ncRNAs, implies that more is yet to come.

## Materials and methods

### Cell culture and iCLIP

P19 cells were cultured as described [13]. For iCLIP, P19 SRSF3-BAC or P19 SRSF4-BAC cells [13] were irradiated with 100 mJ/cm^2 ^UV light. The iCLIP was performed as described [15]. Protein G Dynabeads coupled with goat anti-GFP antibody (a kind gift from D Drechsel, MPI-CBG, Dresden) were used for the immunopurification. The recovered RNA was reverse transcribed into cDNA. After size-purification and amplification the cDNA was subjected to high-throughput sequencing by Illumina Genome Analyser II (single-end 32-nucleotide reads). The adapter oligonucleotides, reverse transcription primers and primers for amplification were as described [15]. A more detailed description is in the Supplementary Methods in Additional file 1.

### Mapping of sequences to mouse genome and sequence analysis

The sequences corresponding to an experiment were identified by a defined barcode and random barcodes were registered. The barcodes were removed before mapping to the mouse mm9 sequence assembly using Bowtie version 0.12.5. Two mismatches were allowed in the mapping, and only CLIP-tags mapping to unique positions were considered. For analysis of significant crosslink sites, the iCLIP positions were randomized. The randomization was done within co-transcribed regions as described [17]. Ensembl59 annotation based on the mouse mm9 genome assembly was used. The statistical approach used to identify significant crosslink sites and CLIP-tag clusters was as described [16,17].

Z-score analysis for enriched pentamers was performed essentially as described [17]. Pentamers were used because they are the longest motifs that could be statically derived from the data. Because we noticed that the inclusion of the actual crosslink nucleotide and the positions immediately surrounding it always resulted in a run of U nucleotides as the most enriched motif (data not shown), we excluded the crosslink site from the analysis and thus avoided bias towards any nucleotide due to differences in crosslinking efficiency. The positions of the crosslinking nucleotide were extended by 30 nucleotides in both directions. Only one occurrence of a pentamer within the evaluated interval [(-30, -10), (10, 30)] relative to each cross-link was counted and each occurrence of a crosslink site was weighted by 1.0. Reference data were generated 100 times by random shuffling of iCLIP crosslink positions within corresponding genome segments (within same genes) and a Z-score was calculated relative to the randomized genomic positions. The top five pentamers were used to calculate the binding consensus motif [53].

### Mapping of crosslink sites to exon-intron junctions and branch points

Crosslink sites located within a maximum 600-nucleotide window [-300,+300] around exon-intron boundaries were mapped to these regions. Each occurrence of a crosslink site was assigned to the closest exon-intron boundary, counted as 1.0 and normalized by number of junctions spanning the crosslink position. In the case of exon-intron junctions, only the last half of the exon and first half of the intron were used to obtain the distribution of exons and introns spanning each position relative to the boundary. Similarly, the last half of introns and the first half of exons were used for intron-exon junctions. Junctions where exons were shorter than 60 nucleotides or introns shorter than 200 nucleotides were ignored (< 15% of all possible junctions). For branch point RNA maps, we used computationally predicted branch points from Corvelo *et al*. [39]. Only the best, non-negative SVM scored branch point that resided in the last half of intron was used. In case of ties, we used the branch point closest to the intron-exon junction. For normalization, only the last half of the intron was used, and the branch point was at position zero. We ignored introns shorter than 240 nucleotides and introns where the branch point was closer than 20 nucleotides to the intron-exon junction.

### Minigene analysis

For the knockdown of *SRSF3 *or *SRSF4*, esiRNA sequences described previously were used [13]. For the overexpression, human *SRSF3 *and *SRSF4 *cDNA were cloned into pYFP-N2 and pEGFP-N3 vectors (Clontech), respectively. To construct the minigenes, the region of interest was PCR amplified from P19 genomic DNA and cloned into pcDNA3 vector (Invitrogen). The plasmids carrying the minigenes were co-transfected together with either *SRSF3 *or *SRSF4 *esiRNA or overexpression construct into P19 cells using Lipofectamine 2000 (Invitrogen). Empty vectors or esiRNA targeting EGFP were used as controls. Twenty-four hours post-transfection, total RNA was isolated using acid phenol-chloroform extraction (Ambion). After DNaseI treatment the RNA was reverse transcribed with Superscript III (Invitrogen). The splicing patterns of minigenes were analyzed using vector-specific PCR primers. To abrogate the nonsense-mediated decay pathway, P19 wt cells were treated with 300 μg/ml cycloheximide for 3 hours. Untreated samples were processed in parallel. Total RNA was extracted and the samples treated as described above. Gene-specific primers spanning the exons flanking the PTC-containing cassette exon were used. Total expression of *SRSF2, SRSF5 *and *SRSF7 *was determined by RT-qPCR using primers spanning an exon-exon junction. *ACTB *was used as a reference gene to normalize for cDNA synthesis efficiency. Primer sequences are available upon request.

### UV crosslinking and RNA immunoprecipitation

UV-crosslinked cells (see above) were harvested and the cytoplasmic fraction was separated. The total cell pellet was suspended in NET-2 buffer (50 mM Tris-HCl pH 7.4, 150 mM NaCl; 0.05% (v/v) Nonidet P-40) for western blot analysis or Trizol (Invitrogen) for RNA isolation. For the fractionation, the cell pellet was suspended in hypotonic buffer (10 mM Hepes, pH 7.4; 10 mM NaCl; 3 mM MgCl_2_; 0.3% (v/v) Nonidet P-40, RNaseOUT; complete protein inhibitor cocktail), the nuclear pellet was collected by centrifugation, and the supernatant was collected as the cytoplasmic faction. The cytoplasmic fraction was used as such or RNA was extracted as above. Input, mock and immunoprecipitation samples were independently reverse primed with either oligo-dT or hexamer primers. Primers specific for individual histone mRNAs were used for qPCR amplification; *POFUT1 *was used as a reference gene for SRSF3 and *DTMYK *for SRSF4 to normalize for cDNA synthesis efficiency. Oligo-dT and hexamer samples were normalized independently of each other to their respective input. The primer sequences are available upon request.

### Database accession

The sequencing data have been submitted to the Array Express database [54], accession number E-MTAB-747.

## Abbreviations

BAC: bacterial artificial chromosome; CHX: cycloheximide; CLIP: crosslinking and immunoprecipitation; EGFP: enhanced green fluorescent protein; GO: Gene Ontology; iCLIP: individual nucleotide-resolution UV cross-linking and immunoprecipitation; lincRNA: long non-coding RNA; mRNP: mitochondrial ribonucleoprotein ; ncRNA: non-coding RNA; NMD: nonsense-mediated decay; ORF: open reading frame; PTC: premature termination codon; RIP: RNA immunoprecipitation; RNP: ribonucleoprotein; RRM: RNA recognition motif; RT-(q)PCR: reverse transcription (quantitative) polymerase chain reaction; scaRNA: small Cajal body-specific RNA; SELEX: systemic evolution of ligands by exponential enrichment; SLBP: stem loop binding protein; snoRNA: small nucleolar RNA; UTR: untranslated region; UV: ultraviolet.

## Competing interests

The authors declare that they have no competing interests.

## Authors' contributions

M-LÄ, MMM and KMN designed the experiments; M-LÄ and MMM performed the experiments; M-LÄ, HB, IH, JU, TC and CG analyzed the data; M-LÄ, MMM and KMN wrote the manuscript. The final manuscript has been read and approved by all the authors.

## Supplementary Material

Additional file 1 **Supplementary Information**. Supplementary Materials and methods, References, Figures S1 to S7 and Tables S1 and S3 to S5. Click here for file

Additional file 2 **Supplementary Table S2**. Genes with significant SRSF3 or SRSF4 crosslink sites. Click here for file
