# Supplementary material for: The RNA-binding landscapes of two SR proteins reveal unique functions and binding to diverse RNA classes
Source: Genome Biol. 2012 Mar 21;13(3):R17. doi: 10.1186/gb-2012-13-3-r17 (PMC3439968; doi:10.1186/gb-2012-13-3-r17)

SUPPLEMENTARY INFORMATION

THE RNA-BINDING LANDSCAPES OF TWO SR PROTEINS REVEAL UNIQUE FUNCTIONS AND BINDING TO DIVERSE RNA CLASSES

Minna-Liisa Änkö[1,4]*, Michaela Müller-Mc Nicoll[1], Holger Brandl[1], Tomaz Curk[3], Crtomir Gorup[3], Ian Henry[1], Jernej Ule[2] and Karla M. Neugebauer[1]*

[1]Max Planck Institute of Cell Biology and Genetics, Pfotenhauerstr. 108, Dresden, 01307, Germany

[2] Laboratory of Molecular Biology, Medical Research Council, Hills Road, Cambridge, CB2 0QH, UK

[3]Faculty of Computer and Information Science, University of Ljubljana, Trzaska cesta 25, Ljubljana, SI-1001, Slovenia

[4]Current address: Australian Regenerative Medicine Institute, Monash University, Wellington Road, Clayton, Melbourne, VIC-3800, Australia

*Co-corresponding authors:

Dr. Karla M. Neugebauer, neugebau@mpi-cbg.de

Dr. Minna-Liisa Änkö, minna-liisa.anko@monash.edu

CONTENTS OF SUPPLEMENTARY INFORMATION

Supplementary Methods

Supplementary References

Supplementary Tables S1-4

Supplementary Table S1 related to Figures 1 and 3

Supplementary Table S3 related to Figures 1 and 3

Supplementary Table S4 related to Figure 5 and 7

Supplementary Table S5 related to Figure 7 and 8

Supplementary Figure Legends

Supplementary Figures S1-7

Supplementary Figure S1 related to Figure 1

Supplementary Figure S2 related to Figures 1 and 3

Supplementary Figure S3 related to Figure 4

Supplementary Figure S4 related to Figure 5

Supplementary Figure S5 related to Figures 6

Supplementary Figure S6 related to Figures 7 and 8

Supplementary Figure S7 related to Figures 7 and 8

SUPPLEMENTARY METHODS

Cell culture and iCLIP

P19 cells were cultured in DMEM (4.5g l^{-1} glucose), supplemented with 10% (v/v) FCS and penicillin/streptomycin under humified 5% CO_2 at 37°C. For iCLIP, P19 SRp20-BAC or P19 SRp75-BAC cells [1] were irradiated with 100 mJ/cm^2 UV light. After cell lysis RNA was partially digested with RNaseI and immunoprecipitation was performed with Protein G Dynabeads coupled with goat anti-EGFP antibody (a kind gift from D. Drechsel, MPI-CBG, Dresden). After immunoprecipitation RNA was labeled on beads with radioactivity at the 5' end and ligated to an adapter oligonucleotide at the 3' end. The RNA-protein complexes were separated by SDS-PAGE and transferred onto nitrocellulose membrane. The RNA-protein complexes were visualized by autoradiography and protein bound RNA was isolated by proteinase K treatment. The recovered RNA was reverse transcribed into cDNA using reverse primers with an adapter sequence. The cDNA was size-purified in a TBE-urea gel, circularized and annealed to an oligonucleotide complementary to the BamHI site incorporated by the reverse primer adapter sequence. The circular cDNA was linearized with BamHI digestion and amplified with primers complementary to the adapter sequences. The amplified DNA was subjected to high-throughput sequencing by Illumina Genome Analyser II (single-end 32 nucleotide reads). The adapter oligonucletides, reverse transcription primers and primers for amplification were as described [2].

SDS-PAGE and Western blot

The specificity and efficiency of the immunopurification was determined by SDS-PAGE and Western blot. The immunopurification was performed as for iCLIP using goat anti-EGFP antibody (a kind gift from Dr. David Drechsel, Dresden, Germany), 5% of the cells extract was kept as an input. Five percent sample was taken from the bead supernatant after the incubation. Samples were separated on 4-12% NuPAGE gels (Invitrogen) and transferred to a nitrocellulose membrane. The membrane was probed with a mouse anti-EGFP antibody (Roche Applied Sciences). To determine the efficiency of SRSF3 or SRSF4 knockdown or overexpression in parallel to the preparation of the RNA samples an aliquot of cells was taken to prepare NET-2 extracts (50 mM Tris-HCl pH 7.4, 150 mM NaCl, 0.05% (v/v) Nonidet P-40, Complete protein inhibitors (Roche Applied Sciences)). Five to 25 µg of total protein was separated as above. The antibodies used were mAb104 [3] against the SR protein family and rabbit anti-GAPDH (FL-335, Santa Cruz Biotechnologies). To quality control the cell fractionation, 5-25 µg protein of the total cell extract, nuclear and cytoplasmic fractions were separated as above. The antibodies used for the detection were anti-GAPDH (FL-335, Santa Cruz Biotechnologies), anti-TRN-SR2 (Abcam) and anti-histone H3 (Abcam).

Computational analysis

Multiple Em for Motif Elicitation (MEME) was used to identify enriched sequences motifs within the scRNAs with crosslink sites. The region with significant CLIP-tag clusters within each scaRNA was extracted, the sequences were aligned and a consensus motif was retrieved. The minimum

motif width allowed was 4 and maximum 25 nucleotides, and only single occurrence within the analysis window was considered.

The Database for Annotation, Visualization and Integrated Discovery (DAVID) v6.7 at http://david.abcc.ncifcrf.gov was used to compute overrepresented functional categories among protein coding genes with significant SRSF3 or SRSF4 CLIP-tag clusters. The Bonferroni correction was used to correct for multiple testing and only molecular function (MF) and biological process (BP) categories were included in the analysis.

Immunocytochemistry

Subconfluent P19 SRSF3-BAC or SRSF4-BAC cells were fixed with 4% (w/v) paraformaldehyde and stained with rabbit goat anti-NPAT antibody (C-19, Santa Cruz Biotechnologies), followed by a species specific secondary antibody (Alexa 568, Invitrogen). DAPI was used to mark the nucleus. The samples were mounted and analyzed with Delta Vision system (Applied Precision).

SUPPLEMENTARY REFERENCES

1. Änkö M-L, Morales L, Henry I, Beyer A, Neugebauer KM: **Global analysis reveals SRp20- and SRp75-specific mRNPs in cycling and neural cells**. *Nat Struct Mol Biol* 2010, **17**:962-970.

2. Konig J, Zarnack K, Rot G, Curk T, Kayikci M, Zupan B, Turner DJ, Luscombe NM, Ule J: **iCLIP reveals the function of hnRNP particles in splicing at individual nucleotide resolution**. *Nat Struct Mol Biol* 2010, **17**:909-915.

3. Roth MB, Zahler AM, Stolk JA: **A conserved family of nuclear phosphoproteins localized to sites of polymerase II transcription**. *J Cell Biol* 1991, **115**:587-596.

SUPPLEMENTARY TABLES

Supplementary Table S1. Mapping information. Repl.=replicate

	**SRSF3** Repl. 1	**SRSF3** Repl. 2	**SRSF3** Repl. 3	**SRSF4** Repl. 1	**SRSF4** Repl. 2	**SRSF4** Repl. 3	**EGFP-NLS** Repl.1	**EGFP-NLS** Repl. 2	**EGFP-NLS** Repl. 3
Reads mapping to the genome	768,977	4,403,140	186,789	387,377	2,227,000	147,869	6,352	1,259	4,866
Reads after random barcode evaluation	346,287	851,676	14,515	136,445	75,708	34,548	713	373	1,525
Crosslinking nucleotides	328,008	744,660	13,957	122,814	64,188	33,452	448	198	1,287
Total reads (barcode evaluated)		1,212,480			243,501			2,611	

Supplementary Table S3. Significant CLIP-tag cluster information. The clustering was performed as described in Methods.

	SRSF3	SRSF4		SRSF3	SRSF4
Sites FDR<0.05	33,458	10,393	Exons with FDR<0.05 sites	822	203
Crosslink site clusters (FDR<0.05)*	7,570	2,716	Introns with FDR<0.05 sites	1,048	607
Genes with FDR<0.05 sites	2,304	1,055	3’UTRs with FDR<0.05 sites	319	140
Protein coding genes with FDR<0.05 sites	1,919	878	5’UTR with FDR<0.05 sites	67	3

*clustering analysis using 15 nt windows

Supplementary Table S4. Enriched GO terms of genes with significant SRSF3 or SRSF4 crosslink sites. Ten most enriched categories are presented. The count represents the number of genes within the category. Bonferroni correction was used to correct for multiple testing to calculate the p-value.

**SRSF3**<br>**GO term**	**Count**	**p-value**	**SRSF4**<br>**GO term**	**Count**	**p-value**
RNA binding	156	$3.64x10^{-23}$	DNA packaging	23	$6.62x10^{-7}$
Nucleotide binding	337	$4.29x10^{-18}$	Nucleosome assembly	28	$5.65x10^{-5}$
mRNA metabolic process	81	$6.23x10^{-15}$	Chromatin assembly	18	$3.74x10^{-5}$
DNA binding	275	$2.08x10^{-13}$	Nucleosome organization	18	$4.63x10^{-5}$
mRNA processing	70	$2.63x10^{-12}$	Protein-DNA complex assembly	18	$4.63x10^{-5}$
Chromosome organization	90	$2.60x10^{-11}$	Chromatin assembly or disassembly	19	$2.31x10^{-3}$
RNA processing	94	$5.92x10^{-11}$	Calmodulin binding	18	$7.332x10^{-3}$
Chromatin organization	75	$1.62x10^{-10}$	Chromosome organization	37	$8.19x10^{-2}$
RNA splicing	56	$4.15x10^{-10}$	Nucleotide binding	134	$2.89x10^{-2}$
Chromatin assembly or disassembly	39	$6.93x10^{-10}$	Chromatin organization	31	$1.1x10^{-1}$

Supplementary Table S5. Splicing related genes with SRSF3 or SRSF4 CLIP-tag clusters.

SRSF3 Gene symbol	Gene name	SRSF4 Gene symbol	Gene name
Bat1a	HLA-B-associated transcript 1A	Casc3	Cancer susceptibility candidate 3
Bcas2	Breast carcinoma amplified sequence 2	Eftud2	Elongation factor Tu GTP binding domain containing 2
Casc3	Cancer susceptibility candidate 3	Esrp1	Epithelial splicing regulatory protein 1
Celf4	Bruno-like 4, RNA binding protein	Hnrnpa2b1	Heterogeneous nuclear ribonucleoprotein A2/B1
Clasrp	CLK4-associating serine/arginine rich protein	Hnrnph1	Heterogeneous nuclear ribonucleoprotein H1
Cwc22	CWC22 spliceosome-associated protein	Luc7l3	Luc7-like 3
Dbr1	Debranching enzyme homolog 1	Prpf40b	Prp40 pre-mRNA processing factor 40b
Ddx46	DEAD box polypeptide 46	Rbm8a	RNA binding motif protein 8a
Ddx5	DEAD box polypeptide 5	Rbm9	RNA binding motif protein 9
Fusip1	FUS interacting protein (serine-arginine rich) 1	Srrm2	Serine/arginine repetitive matrix 2
Hnrnpa1	Heterogeneous nuclear ribonucleoprotein A1	Snrnp70	Small nuclear ribonucleoprotein 70 (U1)
Hnrnpa2b1	Heterogeneous nuclear ribonucleoprotein A2/B1	Snrpb	Small nuclear ribonucleoprotein B
Hnrnpc	Heterogeneous nuclear ribonucleoprotein C	Snrpf	Small nuclear ribonucleoprotein polypeptide F
Hnrnph1	Heterogeneous nuclear ribonucleoprotein H1	Sfpq	Splicing factor proline/glutamine rich
Hnrnpk	Heterogeneous nuclear ribonucleoprotein K	Srsf4	Splicing factor, arginine/serine-rich 4 (SRp75)
Khsrp	KH-type splicing regulatory protein	Srsf12ip1	SRSF12-interacting protein 1
Lsm1	LSM1 homolog, U6 small nuclear RNA associated	Zcrb1	Zinc finger CCHC-type and RNA binding motif 1
Luc7l3	Luc7-like 3	Zranb2	Zinc finger, RAN-binding domain containing 2
Magoh-rs1	Mago-nashi homolog B		
Ncbp2	Nuclear cap binding protein subunit 2		
Nono	Non-POU-domain-containing, octamer binding protein		
Pabpc1	Poly(A) binding protein, cytoplasmic 1		
Prmt5	Protein arginine N-methyltransferase 5		
Prpf38b	PRP38 pre-mRNA processing factor 38 domain containing B		

Prpf40a	PRP40 pre-mRNA processing factor 40a	
Prpf8	Pre-mRNA processing factor 8	
Ptbp1	Polypyrimidine tract binding protein 1	
Ptbp2	Polypyrimidine tract binding protein 2	
Rbm39	RNA binding motif protein 39	
Scaf1	SR-related CTD-associated factor 1	
Sf1	Splicing factor 1	
Sf3a1	Splicing factor 3a, subunit 1	
Sf3a2	Splicing factor 3a, subunit 2	
Sf3b1	Splicing factor 3b, subunit 1	
Sf3b3	Splicing factor 3b, subunit 3	
Sf3b4	Splicing factor 3b, subunit 4	
Sfpq	Splicing factor proline/glutamine rich	
Snrnp35	Small nuclear ribonucleoprotein 35 (U11/U12)	
Snrnp48	Small nuclear ribonucleoprotein 48 (U11/U12)	
Snrpb	Small nuclear ribonucleoprotein B	
Snrpf	Small nuclear ribonucleoprotein polypeptide F	
Snrpg	Small nuclear ribonucleoprotein polypeptide G	
Srrm2	Serine/arginine repetitive matrix 2	
Srsf1	Splicing factor, arginine/serine-rich 1 (ASF/SF2)	
Srsf2	Splicing factor, arginine/serine-rich 2 (SC35)	
Srsf3	Arginine/serine-rich 3 (SRp20)	
Srsf5	Splicing factor, arginine/serine-rich 5 (SRp40)	
Srsf7	Splicing factor, arginine/serine-rich 7 (9G8)	
Tardbp	TAR DNA binding protein	
Thoc4	THO complex 4	
Tra2a	Transformer 2 alpha	
Tra2b	Transformer 2 beta	
U2af1	U2 small nuclear ribonucleoprotein auxiliary factor 1	
U2af2	U2 small nuclear ribonucleoprotein auxiliary factor (U2AF) 2	
Ybx1	Y box protein 1	
Zranb2	Zinc finger, RAN-binding domain containing 2	

SUPPLEMENTARY FIGURE LEGENDS

Supplementary Figure S1. Characterization of iCLIP samples. (**A**) Specificity and efficiency of goat anti-EGFP immunopurification (IP) in P19 SRSF3-BAC and SRSF4-BAC cells. Five percent of the cell lysate as input, 5% of the supernatant after IP and complete eluate were loaded. The expected size of SRFS3-EGFP 65 kDa and SRSF4-EGFP 116 kDa. Mouse anti-EGFP antibody was used for detection. Parental P19 cells (wt) were used as a negative control. (**B**) Autoradiogram of P[32]-ATP labelled protein-RNA complexes after immunopurification. The boxes mark the regions that were cut out and used for RNA isolation. The complete lane was cut out from EGFP-NLS samples because no signal could be detected. (**C**) TBE-urea gel of the amplified iCLIP sequencing libraries. The cDNA was size fractionated before amplification to 60-80 nucleotide (lanes 1 and 4), 80-150 nucleotide (2 and 5) and 150-300 nucleotide (3 and 6) fractions and amplified separately. The 80-150 nucleotide libraries were submitted to next-generation sequencing. M=marker, -RT=no reverse transcriptase control. (**D**) The density of CLIP-tags within a gene and the corresponding expression level in P19 cells based on whole mouse genome microarray data [1] were compared. Upper panel SRSF3 iCLIP performed in P19 SRSF3-BAC cells and lower panel SRSF4 iCLIP in P19 SRSF4-BAC cells.

Supplementary Figure S2. SRSF3 and SRSF4 CLIP-tags cluster to distinct positions in mouse RNAs. Overview of SRSF3 and SRSF4 CLIP-tags and clusters in the chromosome 11. Labels as in Figure 1A.

Supplementary Figure S3. SRSF4 binds to scaRNAs. (**A**) Non-coding RNA *MALAT1* with abundant SRSF3 and SRSF4 CLIP-tag clusters. Labels as in Figure 1A. Note that the genes in the antisense strand run from right to left. (**B**) SRSF3 and SRSF4 CLIP-tags and clusters in two scaRNA genes. (**C**) The CLIP-tag clusters derived from SRSF4 target scaRNAs were aligned using MEME algorithm. The SRSF4 consensus sequence was found in all scaRNAs with SRSF4 CLIP-tag clusters.

Supplementary Figure S4. SRSF3 and SRSF4 bind to histone mRNAs. (**A**) SRSF3 and SRSF4 CLIP-tags and clusters in *HIST1H1C, HIST1H2AE* and *HIST3H2A* genes. Labels as in Figure 1A. The orange arrow heads mark the cleavage site at the 3'UTR. (**B**) Characterization of the cytoplasmic fractionation by Western blot (left panel), RT-PCR and TBE-urea gel (right panel). Antibodies used were anti-histone H3 antibodies for the nuclear fraction, anti-GAPDH and anti-TRN-SR2 antibodies for the cytoplasmic fraction. Primers detecting specifically spliced and unspliced forms of *ACTB* mRNA were used for RT-PCR. The distribution of the tRNA was analyzed by TBE-urea gel. T=total cell extract, N=nuclear fraction, C=cytoplasmic fraction.

Supplementary Figure S5. Pentamer mapping reproduces the RNA map at the exon-intron and intron-exon boundaries. (**A**) Non-normalized RNA maps used to calculate the normalized RNA maps in Figure 6. (**B**) Distribution of exon-intron and intron-exon junctions used for the normalization of RNA maps. The normalization factor corrects for the differences in exon and intron lengths. (**C**) Ten top pentamers identified for SRSF3 (left panel) and SRSF4 (right panel)

were mapped to the 5′ and 3′ splice sites of genes with SRSF3 and SRSF4 CLIP-tag clusters, respectively. The position 0 (dotted line) represents the splice site.

Supplementary Figure S6. SRSF3 binds to NMD-associated cassette exons in SR proteins. (**A**) SRSF3 and SRSF4 CLIP-tags and clusters around the alternative cassette exon of *SRSF5*, *SRSF2* and *SRSF7* genes. Labels as in Figure 1A. Note that the genes in the antisense strand run from right to left.

Supplementary Figure S7. SRSF3 controls the level of SR proteins through splicing regulation. (**A**) The efficiency of SRSF3 and SRSF4 overexpression and depletion in P19 cells. Antibody recognizing a common phospho-epitope of the SR protein family members (mAb104) was used for detection. anti-GAPDH antibody was used to control for equal loading. (**B**) The splicing products of *SRSF3* and *SRSF7* minigenes determined after 24 h depletion of SRSF3, SRSF4 or EGFP (ctrl). The alternative exons are marked with light grey. (**C**) The splicing products of *SRSF2* and *SRSF5* minigenes determined after 24 hours overexpression of SRSF3, SRSF4 or EGFP (ctrl). The alternative exons and intron retention events are marked with light grey. (**D**) The expression level of mature *SRSF2*, *SRSF5* and *SRSF7* mRNAs upon EGFP, SRSF3 or SRSF4 overexpression (24 hours) in cell treated with cycloheximide for 3 hours as measured by quantitative PCR. Error bars as SD.

Supplementary Figure S1.

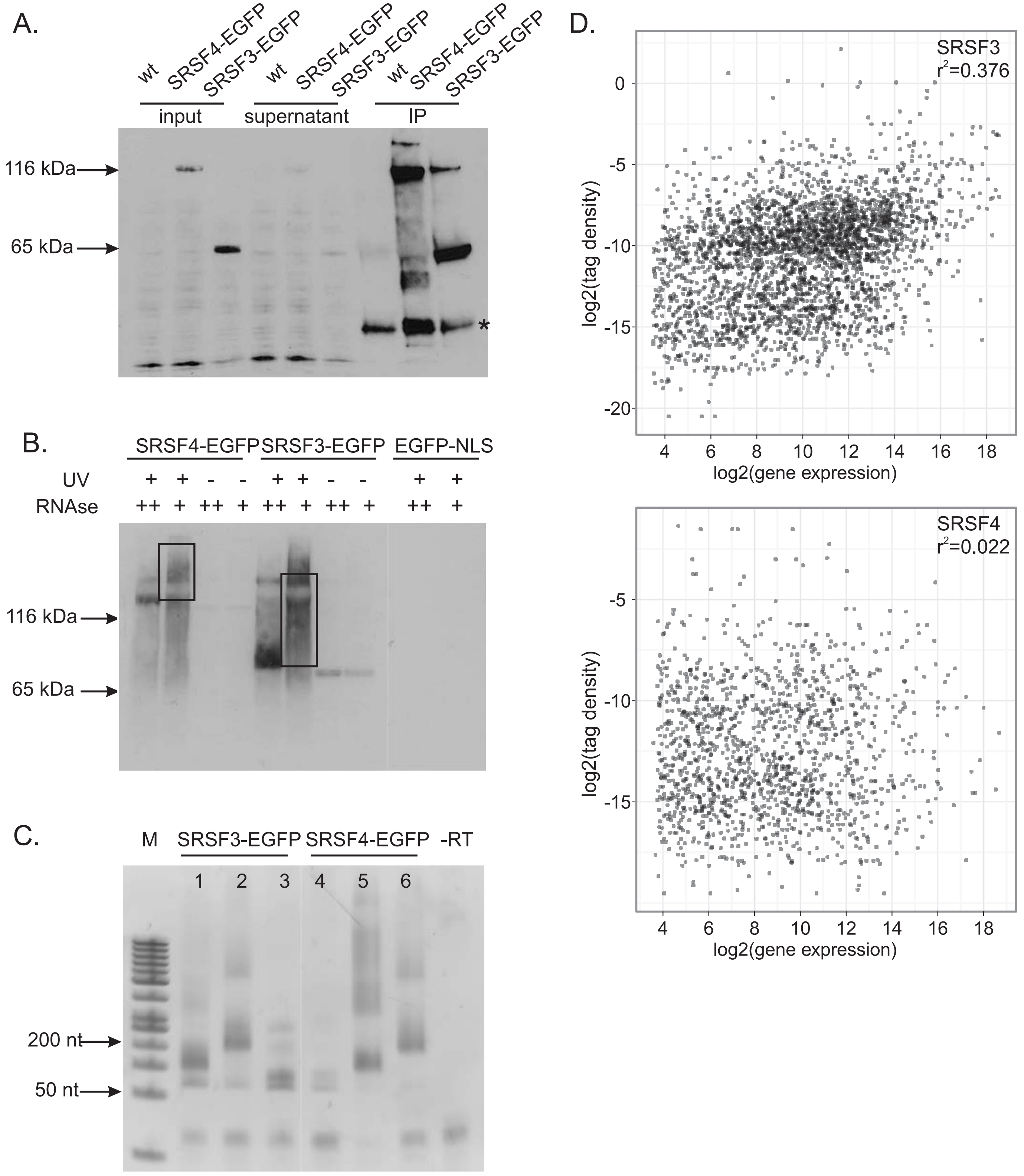

Supplementary Figure S2.

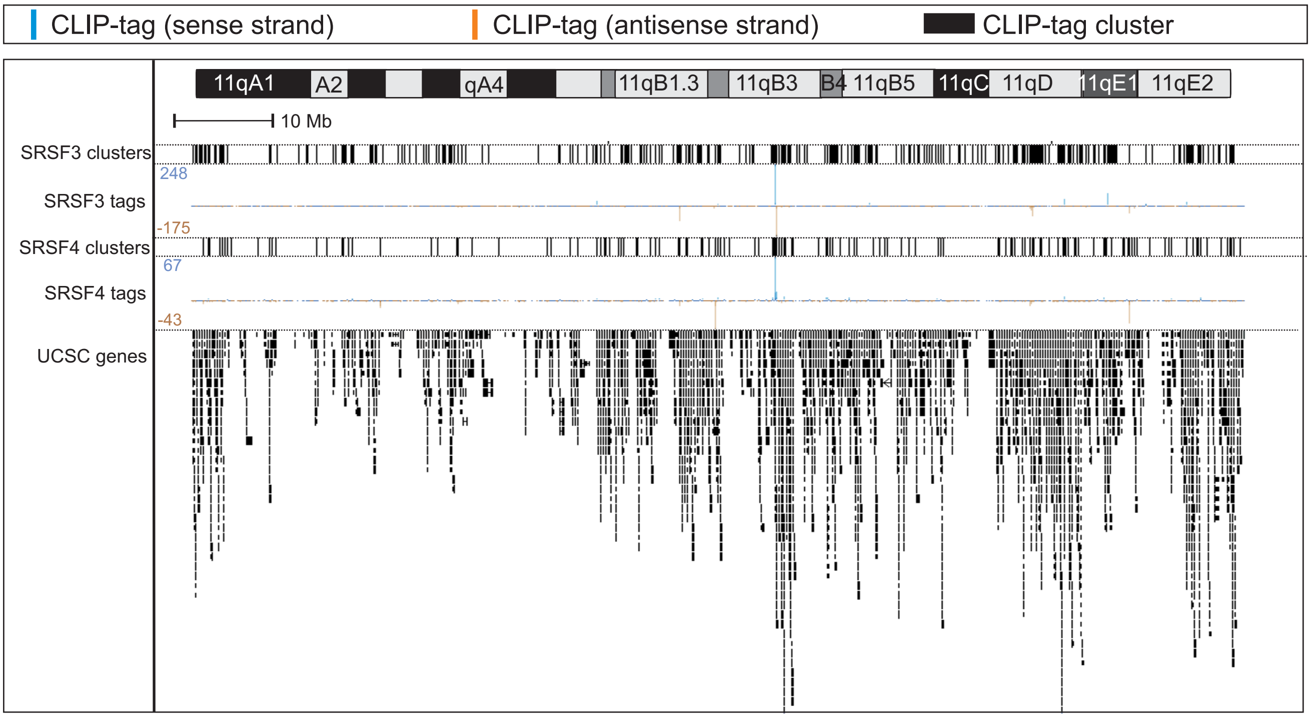

Supplementary Figure S3.

A.

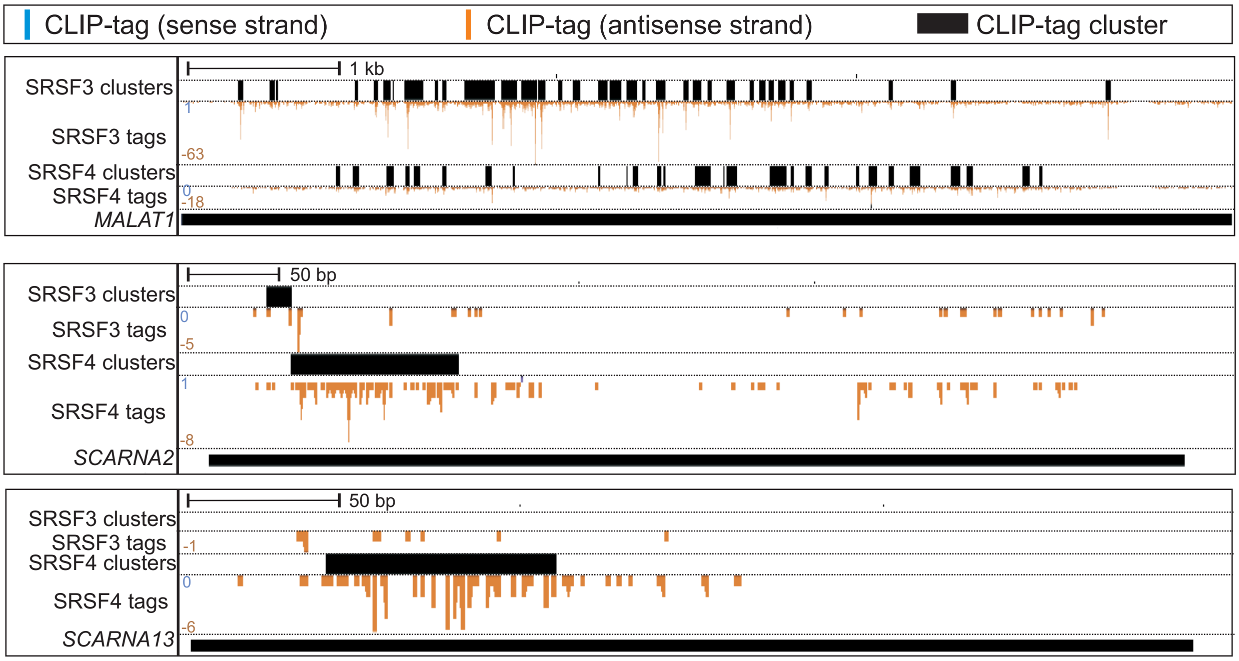

C.

SRSF4

Gene name	Consensus	p-value
SCARNA6	GUUGUUGGG AGUGCU GGCGUCCUUG	4.86×10^{-4}
SCARNA12	GCUGCCACAC AGUACU GGCAGCAUCC	7.23×10^{-4}
SCARNA9	AAAAGGUUUU AGUACU GAAUUUUGUC	7.23×10^{-4}
SCARNA10	GUAGGAAC AGUCUU AUAUUCGAGG	1.28×10^{-3}
SCARNA1	AAAAGCCAUG AGUCUU UUAGGCCCAG	1.28×10^{-3}
SCARNA17	AUCUG AGUUCU GGG	1.79×10^{-3}
SCARNA11	AC AGUGAU UUCCCUCUCA	2.33×10^{-3}
SCARNA2	GGCGCUCAGA AGUGAU GAAUUGAUCA	2.33×10^{-3}
SCARNA15	UGAAGCA AGACUU	4.12×10^{-3}
SCARNA16	CCCUGGGGUU AGAAUU UGCAGUAACA	4.65×10^{-3}
SCARNA13	UUGGCAGUCA AGAUAU UGUUUAGCAG	6.21×10^{-3}
SCARNA7	AAACCCAAAA ACAGCU CACCUAAGUA	8.62×10^{-3}

A.

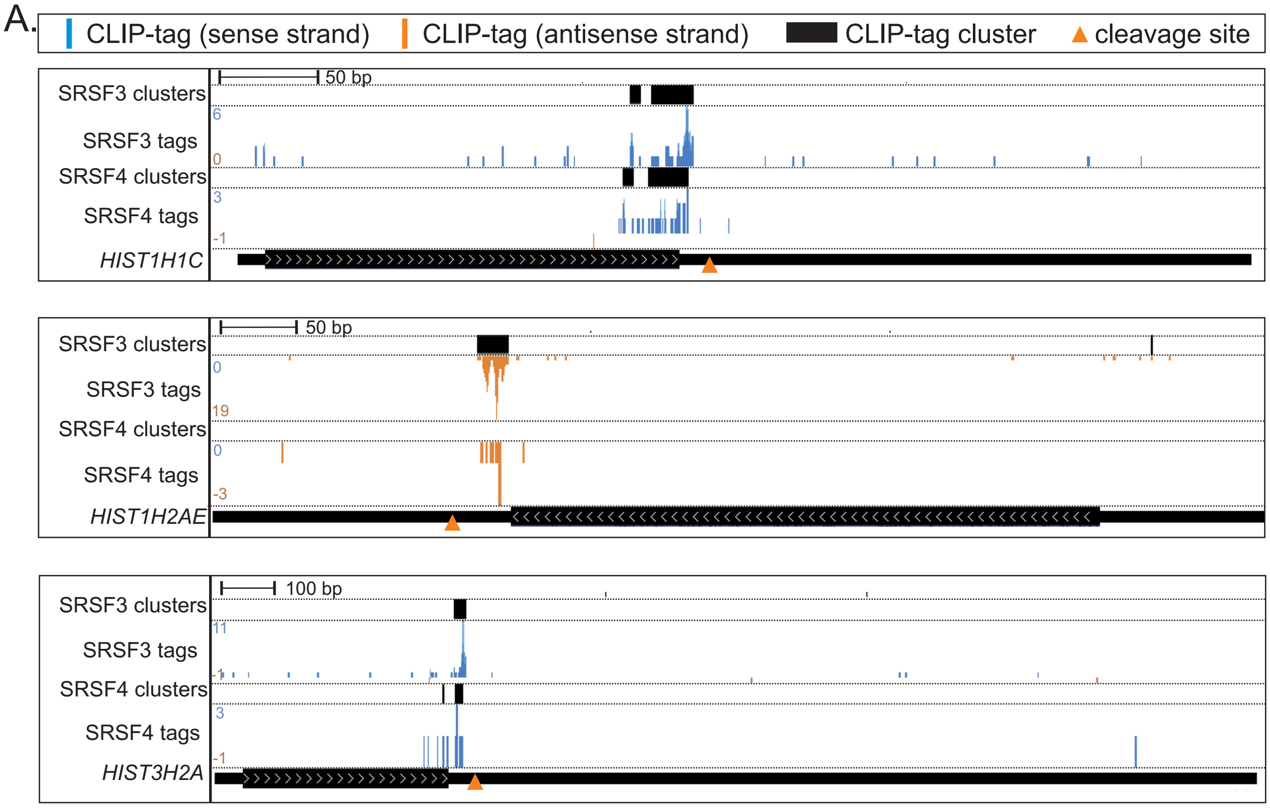
CLIP-tag (sense strand)
CLIP-tag (antisense strand)
CLIP-tag cluster
cleavage site
50 bp
SRSF3 clusters
SRSF3 tags
SRSF4 clusters
SRSF4 tags
HIST1H1C
50 bp
HIST1H2AE
100 bp
HIST3H2A

B.

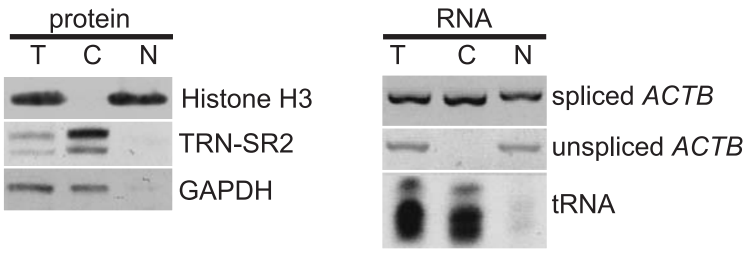
protein
T C N
Histone H3
TRN-SR2
GAPDH
RNA
T C N
spliced ACTB
unspliced ACTB
tRNA

Supplementary Figure S5.

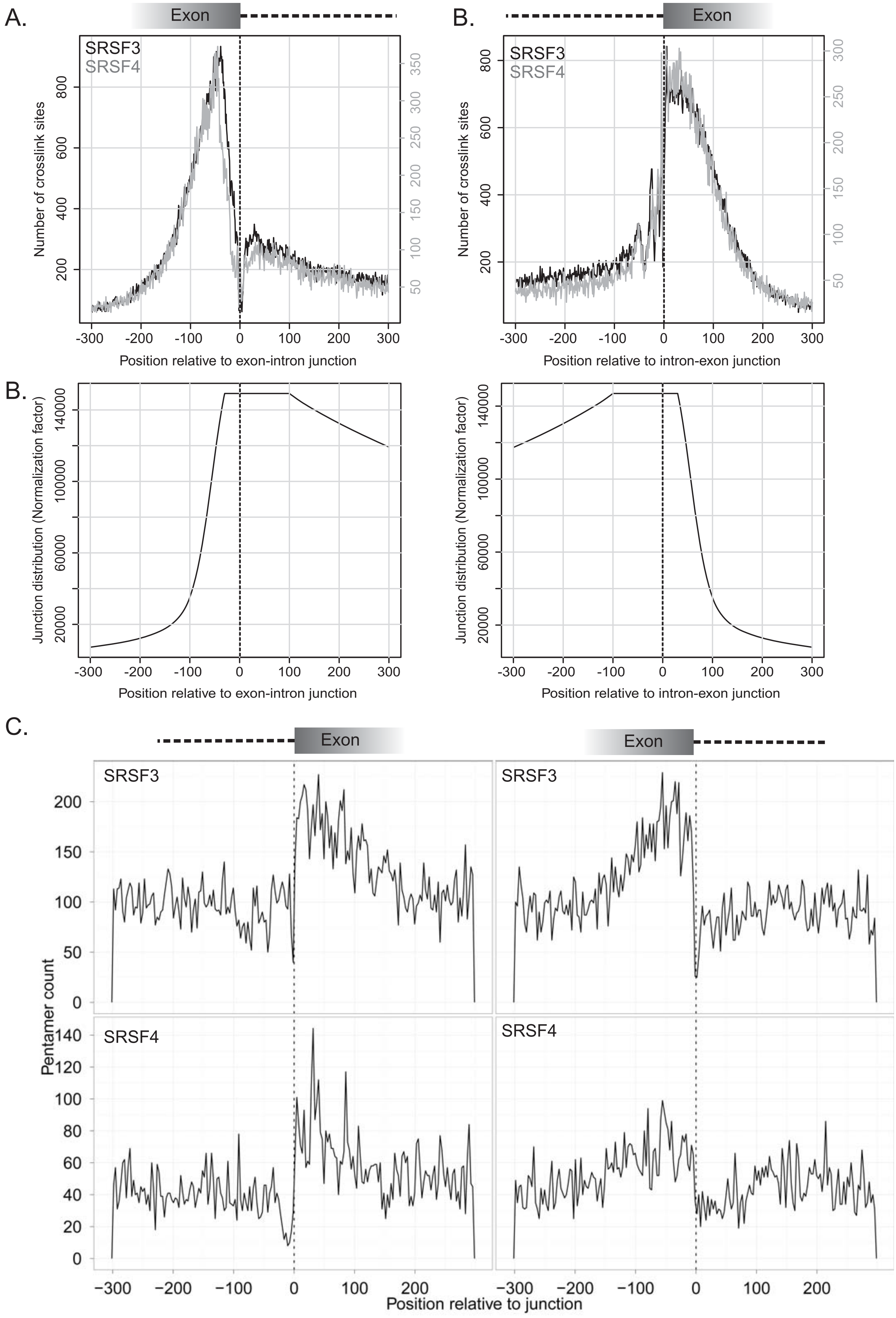

Supplementary Figure S6.

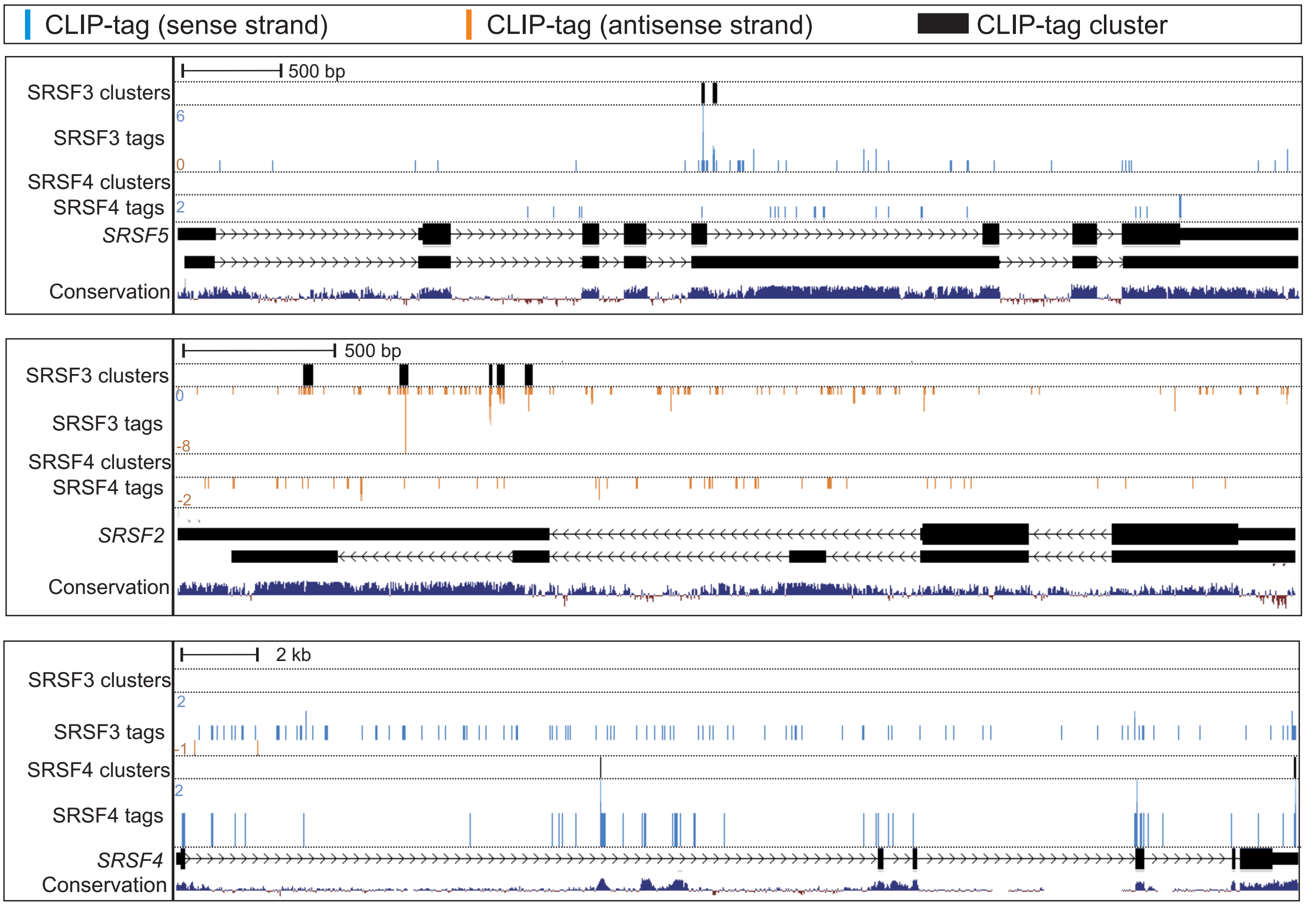

Supplementary Figure S7.

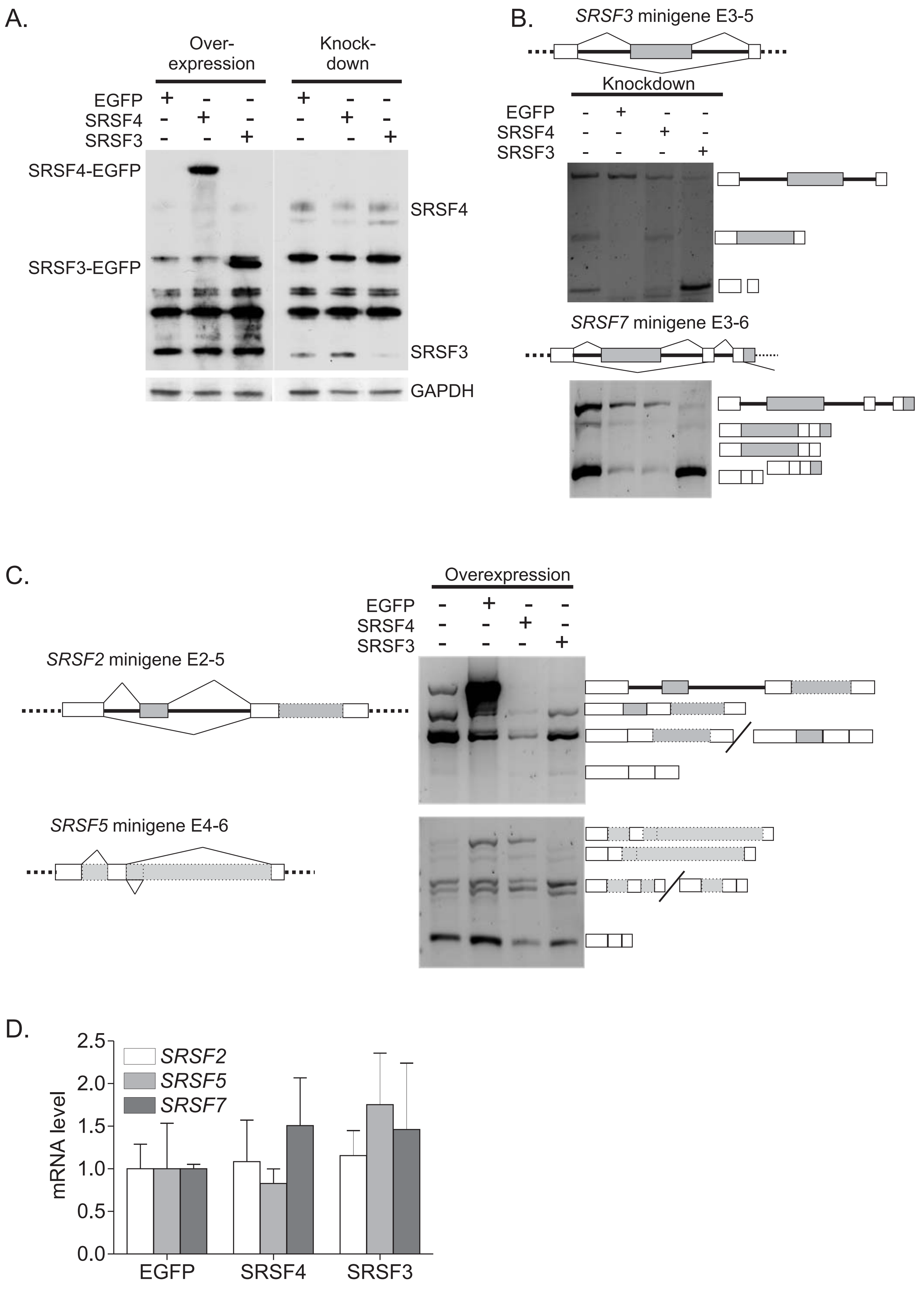

Supplement: Additional file 1 — Supplementary Information. Supplementary Materials and methods, References, Figures S1 to S7 and Tables S1 and S3 to S5. [file gb-2012-13-3-r17-S1.PDF]
